# Nutritional enrichment of black soldier fly larvae towards a sustainable protein food supplement for livestock and poultry production

**DOI:** 10.1016/j.clfs.2026.100042

**Published:** 2026-06

**Authors:** Subbareddy Mekapothula, Bridget Ristow, David Stanford-Beale, Ashraf Alkhtib, Dawn Scholey, Thomas Stringer, Emily J. Burton, Gareth W.V. Cave

**Affiliations:** a School of Science & Technology, Nottingham Trent University, Nottingham, NG11 8NS, United Kingdom; b FlyBox, The Royal Institution, 21 Albemarle Street, London, W1S 4BS, United Kingdom; c School of Animal, Rural & Environmental Sciences, Nottingham Trent University, Southwell, NG25 0QF, United Kingdom

**Keywords:** Black soldier fly larvae, Nanotechnology, Biofortification, Waste management, Nutrition, Circular economy

## Abstract

Black soldier fly larvae (*Hermetia illucens,* BSFL) offer a hyper local, low-carbon sustainable, circular bioeconomic pathway for organic waste valorization and the production of alternative protein sources for livestock and poultry. This study presents a nanomineral biofortification strategy to enhance the nutritional profile of BSFL reared on two distinct feed substrates: conventional industry-standard feed (wheat bran and layer mash) and brewery spent grains. Monodisperse nanoparticles of zinc oxide, manganese oxide, copper oxide and elemental selenium were synthesized using a spinning disc reactor and incorporated into alginate gels (0.25-5 % w/v) and aqueous dispersions (0.05-0.1% w/v), to fortify the industry-standard feed and brewery spent grains. Proximate analysis showed that nanomineral supplementation significantly improved BSFL protein content (up to 40.8%) and fat levels (up to 30.3%), while modulating fiber and ash composition. Elemental analysis revealed that mineral bioaccumulation in BSFL was influenced by both substrate type and nanoparticle formulation. Alginate gels promoted zinc and selenium uptake, whereas aqueous dispersions enhanced manganese and copper accumulation. Notably, BSFL reared on fortified brewery spent grains exhibited higher zinc and manganese accumulation compared to those reared on conventional feed highlighting brewery spent grains potential as a superior base substrate for targeted mineral enrichment. Multi-element nanomineral mix treatments showed no antagonistic interactions, indicating synergistic uptake potential. BSFL grown on fortified brewery spent grains demonstrated enhanced bioconversion efficiency, effectively transforming low-value agro-industrial waste into high-quality insect biomass. The accumulated zinc in BSFL was more bioavailable to poultry than a standard zinc oxide feed additive in the completed broiler feed. This scalable approach enables an adoption tipping point for this emerging alternative protein solution by reducing the cost of production of BSFL and adding value to the insect meal end product.

## Graphical abstract

**Figure:**
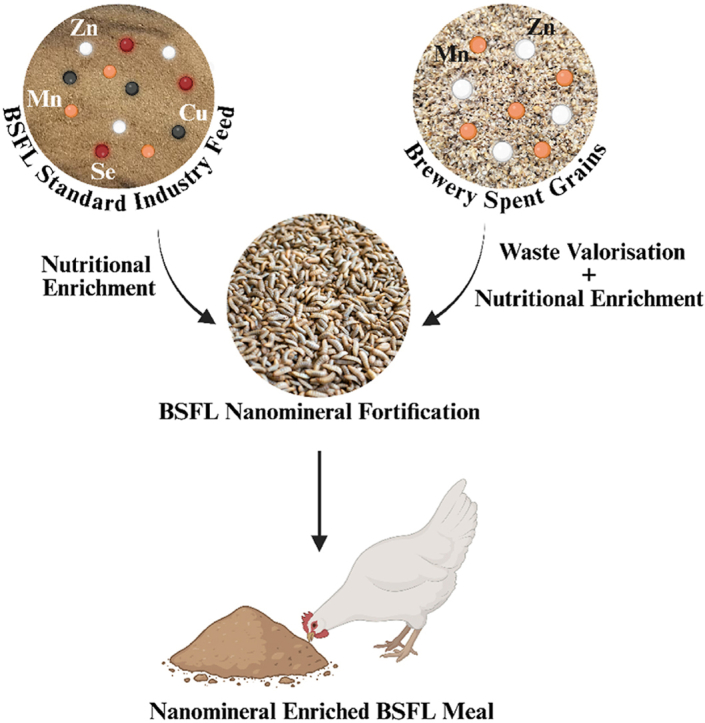

## Abbreviations

BSFL: black soldier fly larvae
WB: wheat bran
LM: layers mash
BSG: brewery spent grains
4DOLs: 4 days of larvae
SDRs: Spinning Disc Reactor

## 1 Introduction

Global population growth, expected to reach 9.8 billion by 2050, is driving a sharp increase in demand for animal protein, with broiler chicken emerging as a dominant source due to its low cost and production efficiency (Pikaar et al., 2018). Reliance on plant-based feed proteins, particularly soybean meal, intensifies land pressure, deforestation and biodiversity loss (Govoni et al., 2021), and with the doubling of feed demand by 2050 (Malila et al., 2024), there is a growing need for low-carbon protein sources, such as microalgae, bacteria, yeasts, and insects (Thornton et al., 2023). As organic waste already accounts for 46% of global solid waste (Bian et al., 2024), circular valorization of these waste systems could help meet net-zero and sustainability targets and recover vital nutrients (Ashokkumar et al., 2022).

**Fig. 1:**
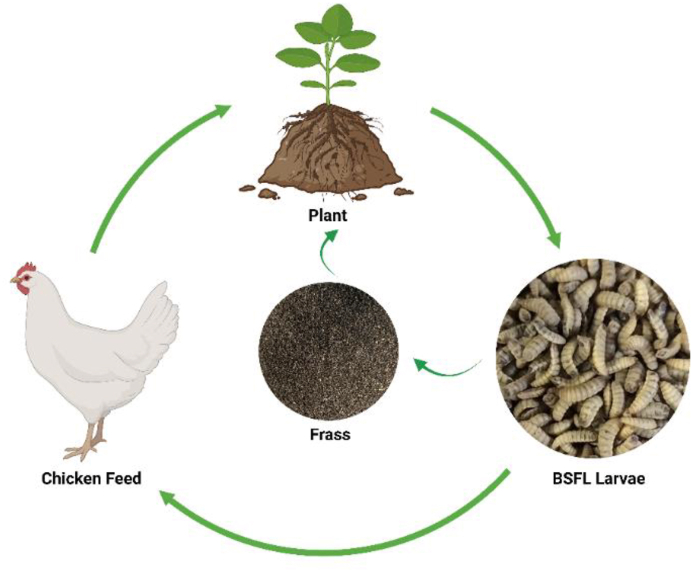
Schematic representation of the circular bioeconomic pathway for Black Soldier Fly Larvae (BSFL) and the recovery of vital nutrients *via* frass fertilizer.

As a result, the search for sustainable, low-carbon alternatives has identified Black soldier fly larvae (BSFL, *Hermetia illucens*) as a scalable solution for circular organic waste bioconversion. The global insect protein market is projected to reach $5.52 billion by 2033, (Edible Insects Market Size) and BSFL is considered an ideal candidate, as it can achieve 50-80% waste-to-biomass conversion (Siddiqui et al., 2022; Mertenat et al., 2018), while generating 47 times less CO_2_ than composting (Fig. 1). (Salomone et al., 2016)

BSFL production utilizes both organic waste for circularity and commercial feeds for nutritional consistency (Danieli et al., 2019a), as substrate nutrient density fundamentally dictates larval development and physiological performance (Barragan‐Fonseca et al., 2018a). Although mineral fortification strategies and optimized feeding regimes enhance biomass quality (Salahuddin et al., 2024; Munguti et al., 2025; Montevecchi et al., 2021), a significant gap remains in developing methods that utilize highly bioavailable mineral sources to maximize the value of BSFL as a low-emission alternative (4.2 t CO_2_e per tonne of BSFL meal) to soybean and fish meal (Macwan et al., 2023; Reyer et al., 2025). While consistent feeds like wheat bran support scalable, regulatory-compliant production, further innovation in targeted mineral enrichment is necessary to realize the full sustainability potential of insect-based ingredients in global food systems (Mlambo et al., 2023).

Nanotechnology provides a transformative approach for mineral fortification of BSFL meal by enabling the use of highly bioavailable mineral nanoparticles in nanoscale forms (1–100 nm) with superior solubility, membrane interaction, and bioavailability compared to conventional salts (McClements, 2020; Hall et al., 2024; Mekapothula et al., 2024). Their large surface area and enhanced cellular uptake significantly improve mineral assimilation in larvae. Using nano-zinc, nano-manganese, nano-copper, and nano-selenium in BSFL substrates is a novel strategy that increases larval growth, mineral deposition, and nutrient density. The resulting nano-fortified BSFL meals deliver more bioavailable minerals to livestock and have been shown to improve digestibility, growth performance, and health in poultry (Alkhtib et al., 2020), aquaculture species (Khan et al., 2020), and ruminants (Almeida et al., 2024). Enhanced mineral uptake also supports metabolic and immune functions, reducing dependence on antibiotics. Overall, nanoparticle supplementation increases the nutritional and commercial value of BSFL and strengthens the sustainability and efficiency of BSFL-based feed production systems by generating a higher-quality feed ingredient from organic waste streams.

To our knowledge, this represents the first report of nanomaterial-biofortified black soldier fly larvae (BSFL) meal. By nano fortifying both commercial industry feeds (wheat bran and layer mash) and agro-industrial waste substrates (brewery spent grains), we reared BSFL with improved growth performance, biomass yield, and nutritional value. We further demonstrate that these nanomaterials, delivered *via* BSFL meal, are bioavailable to broiler poultry. This positions insect meal as a low-emission, alternative protein source. It also reduces production costs and increases the value of BSFL meal for incorporation into complete feeds for poultry and other livestock, helping meet the rising demand for sustainable feed ingredients in the insect-farming industry.

## 2 Materials and methods

### 2.1 Synthesis of nanomaterials loaded alginate gels and characterisation

Briefly, metal oxide nanoparticles (ZnO, CuO, Mn_3_O_4_, and Se) were synthesized using a spinning disc reactor (SDR) by simultaneously pumping aqueous metal salt and sodium hydroxide solutions (60 mL min^−1^) onto a rotating disc (15 cm diameter, 1000-1200 rpm), enabling rapid mixing and precipitation (Cave, 2021). Specific conditions included zinc chloride (1M) with NaOH (3M) for ZnO, copper(II) chloride (1M) with NaOH (2M) at 90 °C for CuO, manganese nitrate (1M) with NaOH (2M) for Mn_3_O_4_, and selenium tetrachloride (0.25 M) reduced by ascorbic acid (0.63 M) in the presence of poly(sodium 4-styrenesulfonate) (2% w/v) for Se. Nanoparticles were filtered, washed, and oven-dried at 120 °C for 3 h before characterisation *via* TEM, SEM and powder X-ray diffraction. Alginate gels were prepared using sodium alginate (4% w/v) in distilled water. For BSFL feed fortification, nanoparticles were formulated into alginate-based gels (4% w/v sodium alginate) at varying concentrations (ZnO: 0.25-5.0%, CuO/Mn_3_O_4_: 0.1-0.5%, Se: 0.06-0.18% w/v) and aqueous dispersions (ZnO: 0.1%, CuO: 0.1%, Mn_3_O_4_: 0.05%, Se: 0.09% w/v) and subsequently were analyzed using ICP-MS for elemental quantification and SEM-EDS for morphology and elemental distribution. Full methodology is provided in supplementary data S3.

### 2.2 BSFL rearing on nanomineral loaded commercial standard feed and brewery spent grains

Briefly, The FlyBox grow container was set to standard operating conditions at 30 °C, 60-65% RH, and 2000 ppmg CO_2_. Commercial feedstock was prepared by hand mixing layers mash (2.4 kg), wheat bran (1.0 kg), and water (6.6 L) to achieve 70 ± 2% moisture, transferred to trays (depth 5 ± 1 cm), and conditioned for 24 h. The brewery spent grain (BSG) diet was formulated using brewery spent grains (8.0 kg), maize (1.0 kg) and water (1 kg) were mixed to achieve the target moisture content (70 ± 2%) and loaded into trays at a uniform depth (5 cm), sourced from Sambrook's Brewery without any preprocessing, milling or particle size reduction. For each 10.0 kg batch, nanomineral alginate gels (ZnO, CuO, Mn_3_O_4_, Se) at a 2.5% (w/w) inclusion rate and aqueous dispersions were incorporated and mixed well. Four-day-old BSFL were sieved from nursery residue and dosed at 7500 larvae per treatment, maintaining equal larval mass across replicates. Trays were stacked to allow airflow and incubated for 8 days under controlled FlyBox grow conditions. At harvest, larvae were separated from frass, weighed for total yield, and immobilized with dry ice for downstream analytical characterisation to measure nutritional content, proximate and moisture analysis. Full methodology is provided in supplementary data S4.

### 2.3 Determination of zinc bioavailability for poultry from BSFL reared on nanozinc fortified substrate

A feeding trial using male, Ross 308 broilers was designed and given ethical approval (WT2409229) to determine the zinc bioavailability (digestibility) of a diet where half the added zinc was provided by insect larvae enriched with nanozinc *via* the substrate enrichment process described previously Birds were reared in 80 by 80 cm pens using the same husbandry and environmental conditions as described in a previous study (Alkhtib et al., 2020). In order to conduct the trial without risking adverse health impacts; the trial was conducted on mid rear (29 day old) broilers with a marginal level of zinc provided *via* the traditional mineral premix route. Birds were reared on a commercial chick starter diet from hatch until day 29, at which point they were transitioned to one of three experimental diets containing distinct zinc sources (Table 1). These treatments consisted of: Diet A (50% of the industry-recommended zinc dose provided *via* a vitamin-mineral premix); Diet B (50% *via* premix and 50% *via* nano-zinc enriched insects); and Diet C (100% of the industry-recommended dose *via* standard premix). Each test diet was fed for a 3-day acclimatization to 8 replicates, with material from 2 chickens fed together pooled to form each replicated. Pairs of ach diet also contained titanium dioxide at 5 g/kg as an inert marker for digestibility measures. Ileal digesta was collected immediately postmortem on day 32 and used to determine dietary zinc digestibility using a standard marker-based method (Alkhtib et al., 2020). Zinc content of blood, excreta, digesta and larvae was carried out using ICP-MS. Using the TiO2 measurements, the zinc digesta and diet values were used to calculate apparent zinc digestibility using the following equation:

$${AD}_{Zn}=1-(\frac{{Zn}_{Digesta}\times M_{Diet}}{{Zn}_{Diet}\times M_{Digesta}})$$

**Table 1:** Dietary treatment formulations for FB01(g/kg).

Raw Material	Diet A	Diet B	Diet C
Wheat	680.2	680.2	679.2
Soybean meal	252.1	252.1	251.1
Soya oil	37.1	37.1	37.1
Salt	2.4	2.4	2.4
Limestone	8.2	8.2	8.2
Dicalcium Phos	4.8	4.8	4.8
Sodium Bicarbonate	2.0	2.0	2.0
Lysine HCL	2.4	2.4	2.4
DL-Methionine	2.6	2.6	2.6
Threonine	1.2	1.2	1.2
Vitmin premix	2.0	2.0	4.0
Insects	-	27.0	-
Titanium dioxide	5.0	5.0	5.0
Analaysed Zn (ppm)	33.0	50.0	49.0

Equation [1] Where AD_Zn_ is the apparent zinc digestibility, Zn is the concentration of zinc (ppm) and M is the concentration of titanium dioxide marker.

Statistical analysis was carried out using SPSS v.28. To determine interactions between the analyzed factors, and one-way ANOVA to test the equality of the means to investigate the effect of dietary treatment on performance.

## 3 Results and discussion

### 3.1 Nutritional analysis of BSFL commercial feed substrates and BSFL life cycle

A well-balanced diet is essential for optimising BSFL growth, survival rate and bioconversion efficiency and overall sustainability. Despite growing interest in BSFL as a sustainable protein source, the nutritional requirements and cost-effective feed formulations remain underdeveloped. Effective mineral fortification depends on understanding nutrient composition from feed and nutrient distribution across larval stages, enabling the design of fortified diets that improve both BSFL's nutritional and bioconversion value. These improvements can help BSFL product viability compared to traditional soya meal and fishmeal in terms of protein and nutritional source, while also offering functional, economic, and sustainability benefits (Danieli et al., 2019b; Barragan‐Fonseca et al., 2018b).

As shown in Table 2, BSFL reared on a commercial standard feed substrate of layer mash (2.4 kg), wheat bran (1.0 kg), and water (6.6 L) (*ca* 70% moisture) under controlled *FlyBox Grow* conditions demonstrated robust growth from 4 to 12 days old. Inoculation with 7500 larvae yielded 1.11 kg of wet biomass (63-65% moisture) and 2.0 kg of frass (32% moisture), with a 98.25% survival rate, 80% substrate reduction and a wet bioconversion rate of 11.74%. An average larval length of 1.48 cm reflects efficient nutrient assimilation and strong adaptability to the feed blend. These results align with FlyBox in-house standard growth parameters and highlight the potential of optimized substrates for insect biomass production.

**Table 2:** Growth performance of black soldier fly larvae reared on commercial standard feed substrates.

BSF Growth Parameters	WBLM feed
Mean length (cm)	1.48 ± 0.1
Survival rate (%)	98.25 ± 0.7
Larval bioconversion (%) (wet mass)	11.74 ± 0.3
Substrate reduction rate (%)	80.70 ± 0.2
Waste reduction index (g/day)	10.09 ± 0.1

**Fig. 2:**
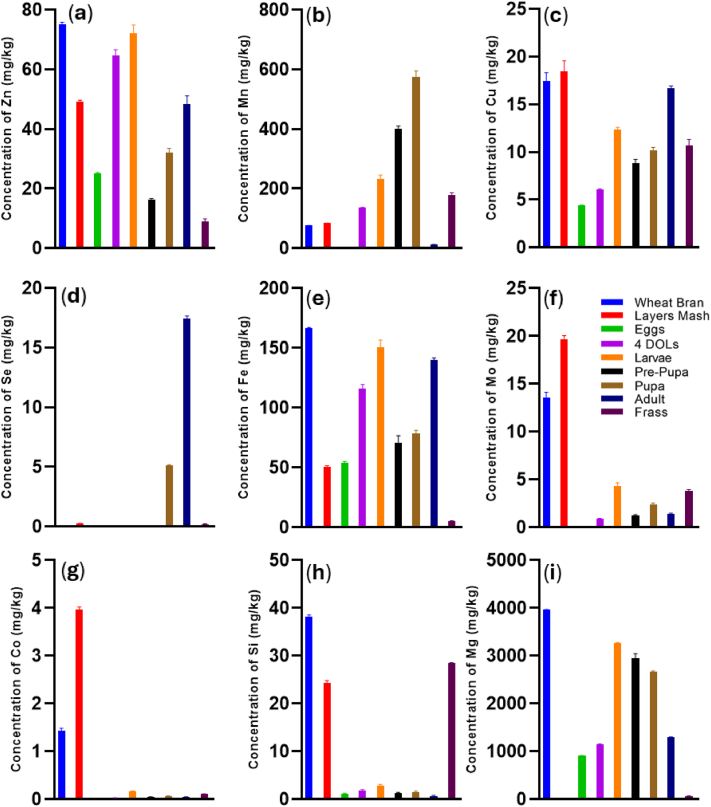
Trace element and mineral concentration (ppm) across commercial industry feed substrates and throughout the BSFL life cycle. Panels show the distribution of (a) Zn, (b) Mn, (c) Cu, (d) Se, (e) Fe, (f) Mo, (g) Co, (h) Si, and (i) Mg within feed components (Wheat Bran and Layers Mash), developmental stages (Eggs, 4 DOLs, Larvae, Pre-pupa, Pupa, and Adult), and frass by-product. Data represent the mean ± standard deviation of triplicate. Correct this if wrongmeasurement*s*In accordance with the European Food Safety Authority (EFSA) and Commission Regulation (EC) No 1831/2003, maximum permissible levels (MPLs) for trace elements in broiler feed are established to ensure safety and nutritional adequacy. These include Fe (450 ppm), Cu (25 ppm), Zn (100 ppm), Mn (100 ppm), Co (1 ppm), I (10 ppm), and Se (0.5 ppm). Elements such as Mg, Ca, and Si are not specifically regulated, offering flexibility in feed formulation. (Regulationa; European Food Safety Authority) Therefore, fortification of BSFL with identified deficient minerals, such as Zn, Mn, Se, and Cu, *via* nanotechnology offers a promising strategy to enhance the nutritional profile and growth performance of larvae. This approach aligns fortified BSFL with EFSA guidelines, improving micronutrient bioavailability in broiler feed while minimizing environmental impact.

The distribution of essential trace elements across feed substrates, wheat bran (WB) and layer mash (LM), and developmental stages of BSFL dynamic physiological processes of uptake, retention, and excretion, are shown in Fig. 2. For instance, zinc present at 125 mg/kg in industry standard feed, peaked at 12 days of larval age (12 DOL), indicating a critical window for nutrient assimilation, followed by a decline during molting and moderate retention in adults, as shown in Fig. 2a. Zinc levels declined during molting because Zn is mobilised for cuticle remodeling, where it functions as a cofactor for chitin-modifying enzymes and other cuticle-associated proteins (Chasapis et al., 2011). The old exuviae retain bound Zn and are shed during ecdysis, resulting in mineral loss. Molting also involves reduced feeding and nutrient absorption, and the rapid post-ecdysis increase in body mass can dilute tissue Zn concentrations. Together, these processes explain the temporary reduction in Zn during BSFL molting (Liu et al., 2017). In BSF adults, Zn is primarily accumulated in intestinal tissues *via* the ZnuCBA transport system and citric acid cycle pathways (Deng et al., 2025). The minimal Zn found in the frass (*ca*.10 mg/kg) suggests efficient assimilation and low environmental loss.

As shown in Fig. 2b, manganese accumulated progressively, peaking in pre-pupal stages, with moderate frass levels indicating partial excretion. Copper and iron showed strong bioaccumulation, particularly in larvae and adults, with low frass concentrations, highlighting their metabolic importance (Fig. 2c and e). BSFL have a greater natural tendency to concentrate minerals than many other insects due to their rapid feeding, high metabolic turnover, and efficient assimilation mechanisms. This makes them suitable for targeted fortification with essential minerals. However, excessively high levels of Cu and Fe can surpass physiological thresholds, leading to oxidative stress, metabolic disruption, reduced growth, and impaired survival, as reported in bioaccumulation studies of undesired substances and in life-cycle mineral analyses. (Deng et al., 2022). Selenium and molybdenum, though present in lower concentrations, were selectively retained during early and mid-development, supporting roles in redox and enzymatic functions (Fig. 2d and f). Magnesium abundant in WB but absent in LM, increased from egg to 12 DOL and was retained through later stages, with moderate frass levels indicating partial excretion, as shown in Fig. 2i. Cobalt and silica showed minimal retention, despite Si's high substrate presence, suggesting poor absorption (Fig. 2g and h).

Subsequently, monodisperse nanominerals of ZnO, Mn_3_O_4_, CuO, and Se were successfully synthesized using a patented continuous flow spinning disc reactor (SDR) (Cave, 2021), which enables precise control over particle size and morphology through optimization of flow rate, disc rotation speed, and surface curvature. As shown from Figures S1-S5, ZnO nanoparticles (55 ± 11.15 nm) exhibited high crystallinity and thermal stability up to 600 °C, with a positive surface charge. Mn_3_O_4_ nanoparticles (80.18 ± 5.41 nm) showed cuboid morphology, excellent phase purity, and thermal stability up to 800 °C, with a negative zeta potential. CuO nanoparticles (18 ± 3.26 nm) were spherical, confirmed as tenorite phase, and thermally stable up to 600 °C, also with a negative surface charge. Se nanoparticles (79.04 ± 7.15 nm) were synthesized *via* reduction with ascorbic acid, displaying good crystallinity and thermal degradation onset at 550 °C. These results demonstrate the SDR's capability to produce reproducible nanominerals with tailored physicochemical properties, suitable for various commercially impactful nutritional enrichment applications for global food security.

For the fortification of BSFL feed substrates to enhance the nutritional profile of BSFL, nano minerals (ZnO, Mn_3_O_4_, CuO, and Se) were formulated into two distinct feed systems, alginate-based hydrogels and aqueous nanoparticle dispersions. Alginate gels were prepared without Ca^2+^ cross-linking agents to prevent undesirable interactions with the Zn, Mn, Cu, and Se nanominerals, which could otherwise form unintended composites and alter their release behavior. The absence of CaCl_2_ results in soft, minimally cross-linked alginate matrices that support controlled diffusion of the nanominerals into the larval feed substrate. This simplified, reagent-free process also provides a cost-effective route for producing nanomineral-loaded gels by reducing both material expenses and processing steps. These systems were systematically evaluated to determine their cost-effectiveness. Scanning electron microscopy (SEM) combined with energy-dispersive X-ray spectroscopy (EDS) was used to characterize sodium alginate gels loaded with ZnO, Mn_3_O_4_, CuO, and Se (w/v). As shown in Figure S6, SEM images revealed distinct surface morphologies for each formulation, ranging from moderately porous (ZnO) and rough (Mn_3_O_4_) to fibrous (CuO) and smooth (Se), while EDS mapping confirmed successful and uniform elemental distribution of the respective metal oxides (Figure S7). Sodium peaks in all spectra validated the alginate matrix. ICP-MS analysis further confirmed high incorporation efficiency and consistency between theoretical and measured nanoparticle concentrations across all gel and dispersion formulations (Table S1). These results demonstrate the structural integrity and compositional uniformity of the gels, supporting their potential for controlled micronutrient delivery in BSFL feed fortification.

### 3.2 Nano fortification of BSFL using commercial industry feed substrates

**Fig. 3:**
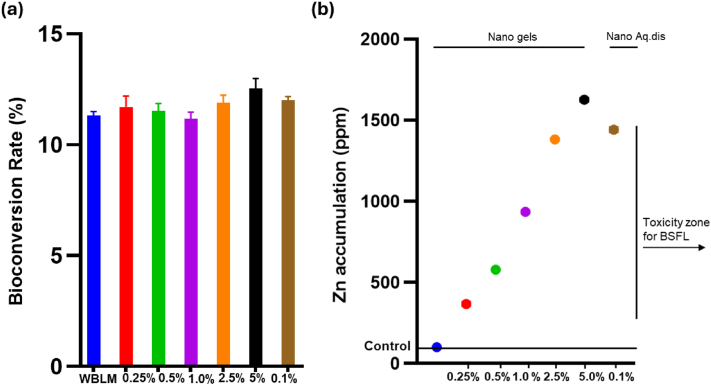
Impact of ZnO nanoparticle (ZnO-NP) delivery systems on the growth performance and zinc accumulation of BSFL (a) Wet bioconversion rate (%) and (b) zinc accumulation (ppm) in BSFL reared on a commercial substrate (wheat bran and layers mash) fortified with ZnO-NP loaded alginate gels (0.25–5.0 % w/v) or aqueous dispersions (0.1 % w/v).

**Fig. 4:**
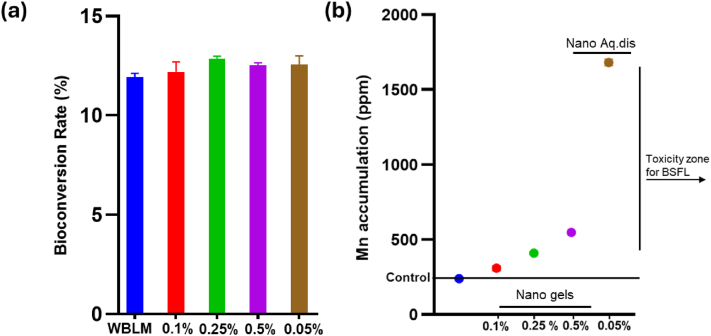
Influence of Mn_3_O_4_ nanoparticle delivery systems on the bioconversion and manganese accumulation of BSFL. (a) Wet bioconversion rate (%) and (b) manganese accumulation (ppm) in BSFL reared on a commercial substrate (wheat bran and layers mash) fortified with Mn_3_O_4_ nanoparticle loaded alginate gels (0.1–0.5 % w/v) or aqueous dispersions (0.05 % w/v).

Zn accumulation increased from baseline values (<100 ppm) to 1626 ppm, representing approximately 16-fold enrichment, indicating significantly enhanced Zn bioaccumulation under optimized nanoparticle delivery. The sharp decline in performance at 5–20% (w/v) suggests a toxicity threshold between 2.5 and 5%, consistent with heavy-metal stress kinetics where ROS generation exceeds the larval antioxidant capacity. The superior performance of alginate gels ≤5% (w/v) suggests that gels modulate Zn release, preventing acute metal overload and supporting steady uptake as show in Fig. 3b. The aqueous dispersion at ≤0.1% w/v showed high uptake efficiency relative to its lower dose, indicating greater ionization and surface reactivity of dispersed ZnO NPs. Bioconversion improvements at optimal gel doses (Fig. 3a) indicate that ZnO may stimulate intermediary metabolism (e.g., TCA cycle enzymes), but only when dosed below the toxicity boundary. The combined gains in bioconversion and Zn load demonstrate that release-controlled matrices (alginate) provide both physiological and functional performance advantages. Subsequently, This allows to create a concentrated Zn supplement that stays within the EFSA regulatory limit of 100 ppmwhen included in complete broiler feed (see Fig. 4).

Mn_3_O_4_ dispersions produced the highest Mn loading (1686 ppm), demonstrating that Mn uptake is concentration-driven and that BSFL possess strong Mn sequestration mechanisms. The ∼50% uptake efficiency reflects a high Mn demand in enzymatic pathways (arginase, Mn-SOD) (Vásquez-Procopio et al., 2019), allowing larvae to absorb and store Mn even when substrate concentrations are increased. Differences between gel (250–1250 ppm) and dispersion uptake patterns indicate differential bioavailability, with dispersions offering readily accessible Mn ions, whereas gels provide controlled release that prevents saturation kinetics. The consistent bioconversion efficiency (12–13%) across all Mn treatments suggests high Mn tolerance, contrasting with the narrower Zn tolerance window, implying Mn-induced oxidative stress thresholds are higher. This reinforces the suitability of Mn fortification for high-load applications. These findings support the use of Mn_3_O_4_ nano-formulations for targeted enrichment in BSFL, with flexibility in the inclusion of Mn fortified BSFL in the animal livestock feed to meet EFSA regulatory limits (≤150 ppm Mn in feed) (Bampidis et al., 2023).

**Fig. 5:**
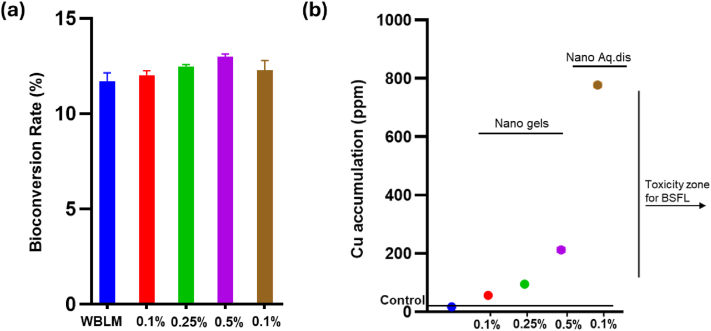
Influence of CuO nanoparticle delivery systems on the bioconversion and manganese accumulation of BSFL. (a) Wet bioconversion rate (%) and (b) manganese accumulation (ppm) in BSFL reared on a commercial substrate (wheat bran and layers mash) fortified with CuO nanoparticle loaded alginate gels (0.1–0.5 % w/v) or aqueous dispersions (0.1% w/v).

As shown in Fig. 5b, CuO dispersions produced the highest Cu loading (778 ppm), indicating that Cu uptake is strongly influenced by nanoparticle solubility and dissolution rate. In contrast, the higher bioconversion efficiency observed in alginate formulations suggests that rapid Cu release from dispersions imposes metabolic costs, likely due to Cu^2+^/Cu^+^ redox cycling and the associated oxidative stress. The dose-dependent increases in Cu uptake in gels reflect controlled assimilation, allowing larvae to avoid copper-induced enzymatic inhibition while still achieving nutritionally relevant enrichment. The resulting trade-off greater Cu accumulation in dispersions versus superior bioconversion in gels, highlights the need to balance bioavailability against oxidative burden when designing Cu fortification strategies.

When BSFL fortified with copper nanominerals were compared with larvae given conventional copper at 50, 500, and 1000 ppm, the nano-copper groups performed better (Zhou et al., 2023). The lower nano-copper doses (25–125 ppmin alginate gels and 666 ppmin nano-suspensions) produced higher growth and greater copper deposition in the larvae. These results agree with earlier findings showing that copper uptake in BSFL depends on both dose and exposure time. Overall, low-dose nano-copper improved copper availability without causing the growth reduction seen at higher conventional copper levels. This mechanistic balance explains why dispersions maximize Cu uptake, whereas gels support more efficient substrate conversion. Furthermore, Consequently, Cu-fortified BSFL produced through optimized formulations can be tailored to remain within the EFSA regulatory limit of 25 ppm Cu in complete feed. (Regulationa)

**Fig. 6:**
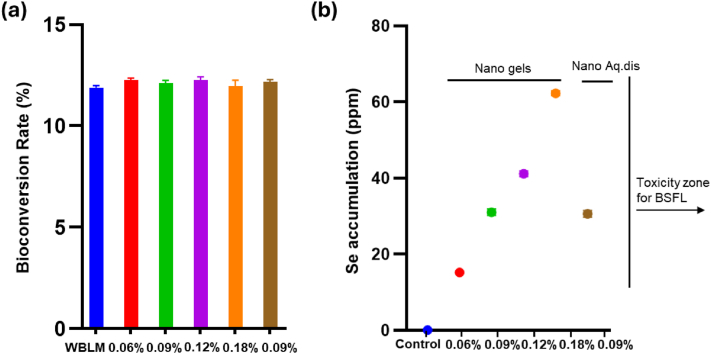
Influence of selenium nanoparticle delivery systems on the bioconversion and manganese accumulation of BSFL. (a) Wet bioconversion rate (%) and (b) manganese accumulation (ppm) in BSFL reared on a commercial substrate (wheat bran and layers mash) fortified with selenium nanoparticle loaded alginate gels (0.06–0.18 % w/v) or aqueous dispersions (0.09% w/v).

Se nanoparticle uptake exhibited clear dose-dependency, with gels achieving ∼62 ppmSe (p < 0.0001), double the aqueous dispersion level as shown in Fig. 6. This indicates that alginate gels enhance Se NP interaction with the gut epithelium or prolong residence time, enabling higher incorporation. The absence of bioconversion penalties suggests that Se-NPs do not trigger acute toxicity at the tested doses, unlike ionic Se sources (e.g., sodium selenite) (Li et al., 2025). The gel-mediated enhancement illustrates that nanoparticle stabilization and controlled release prevent Se detoxification pathways (e.g., methylation, excretion), resulting in improved assimilation. The effective enrichment without performance loss positions Se-NP gels as a superior fortification method. The Se enriched BSFL can be integrated into the broiler chicken feed to meet European Union regulatory limit of 0.5 ppmcomplete feed (Bampidis et al., 2024).

**Fig. 7:**
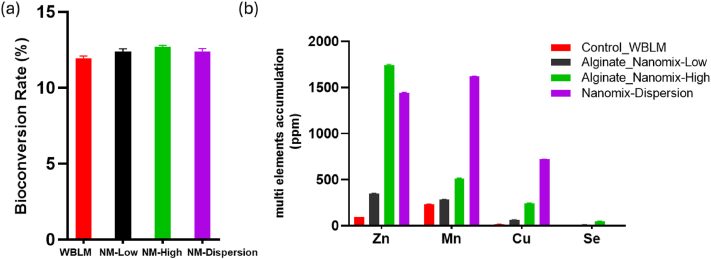
Wet bioconversion rate of BSFL (a) and Zn, Mn, Cu, and Se nano accumulation in BSFL from nanomix alginate gels and. Influence of multi-element nanomineral mix (Nanomix) formulations on the bioconversion and multi-element accumulation of BSFL. (a) Wet bioconversion rate (%) and (b) cumulative mineral accumulation (ppm) for Zn, Mn, Cu, and Se in BSFL reared on nanomix alginate gels and nanomix dispersion fortified commercial industry feed.

As shown in Fig. 7, high-dose alginate gels produced the best bioconversion, indicating that multi-element gels reduce competitive ion absorption, enabling balanced uptake without metabolic overload. In contrast, the higher Mn and Cu accumulation in dispersions aligns with their higher ionic reactivity, supporting more aggressive uptake but at potentially greater physiological cost. Zn's preferential uptake in gels indicates that Zn absorption benefits from slower, sustained release, preventing transporter saturation or competition with Mn and Cu. The observed increase in Zn uptake in the presence of Mn and Cu suggests no antagonistic interactions were observed, with formulation-dependent uptake patterns persisting in the multi-element mix. Se maintained dose-responsive uptake regardless of formulation, indicating that Se transport pathways are less competitive than those of Zn, Mn, or Cu. These patterns confirm that mixed-nanomineral systems require formulation-specific optimization to manage element-element interactions and maximize efficiency.

Proximate analysis of BSFL fortified with nanomaterials on commercial feed substrates shows increases in both protein and fat across all nano-fortified groups. These improvements suggest that nanoparticle treatments enhanced metabolic efficiency. This effect is likely driven by improved micronutrient cofactor availability, supporting more effective protein synthesis and lipid metabolism. The higher ash content in alginate-gel groups reflects superior mineral retention, consistent with slower, matrix-mediated release. Reduced fiber content indicates enhanced digestion or substrate breakdown efficiency, aligning with observed improvements in bioconversion. The small but consistent difference, dispersions improving protein slightly more, gels improving mineral retention more demonstrates that physicochemical behavior of nanoparticles modifies both nutrient uptake and metabolic allocation pathways.

### 3.3 Nano fortification of BSFL using brewery spent grains

Brewers’ spent grain (BSG), which makes up approximately 85% of brewery solid waste and exceeds 36 million t annually, is highly moist and prone to rapid microbial spoilage, limiting its use and often leading to low-value disposal (Nyhan et al., 2023; Eliopoulos et al., 2021). Its landfilling contributes substantial emissions (∼513 kg CO_2_ eq per ton) highlighting the environmental burden and the urgent need for sustainable valorization strategies. (Lalić et al., 2025). Converting BSG through black soldier fly larvae (BSFL) offers a scalable circular-bioeconomy solution, transforming this nutrient-rich by-product into high-value protein, lipids, and frass fertilizer (Resconi et al., 2024; Peguero et al., 2024). This approach aligns with EU Regulation 2017/893 permitting insect protein in feed and supports broader EU sustainability and Farm-to-Fork objectives by reintegrating industrial by-products into low-carbon food and agriculture systems. (Regulationb; Farm to fork strategy)

**Fig. 8:**
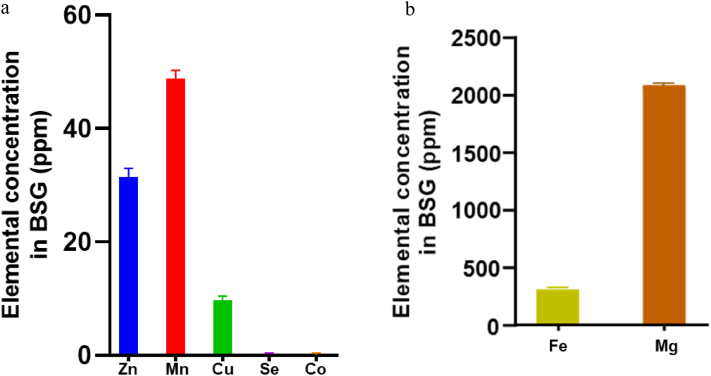
Elemental composition (ppm) of brewery spent grains substrate. (a) Concentrations (ppm) of Zn, Mn, Cu, Se, and Co; and (b) concentrations (ppm) of Fe and Mg.

Elemental analysis of BSG showed that it contains recoverable minerals relevant to BSFL nutrition and frass fertilizer production. As shown in Fig. 8, Mg was the most abundant element (2200 ppm), followed by Fe (250 ppm), with moderate levels of Mn (50 ppm), Zn (33 ppm), and Cu (10 ppm), and only trace amounts of Co and Se (0.2 ppmeach). All concentrations were below EU regulatory limits for animal feed (Zn 120 ppm, Mn 100 mg/kg, Cu 10 ppm, Co 10 ppm, Se 2 ppm), indicating that BSG is safe but mineral-deficient, particularly in Zn and Mn. To overcome these limitations, BSG was used as both a valorization substrate and a carrier for nano-fortification, replacing conventional commercial feedstocks such as wheat bran and layer mash. Supplementing BSG with Zn and Mn nanoparticles enabled targeted enhancement of mineral availability while improving BSFL bioconversion performance, providing a sustainable route for upgrading this underutilized agro-industrial by-product.

**Fig. 9:**
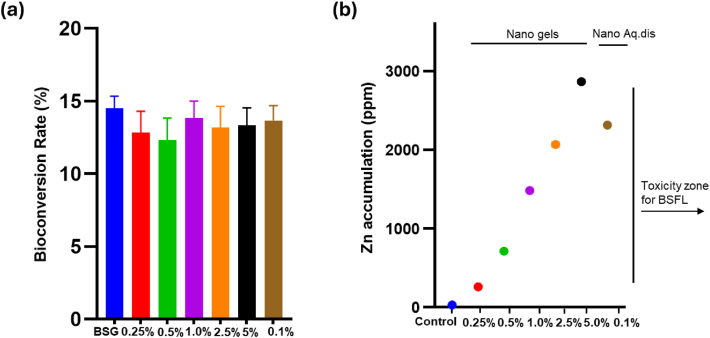
Influence of ZnO nanoparticle delivery systems on the bioconversion and zinc accumulation of BSFL reared on brewery spent grains. (a) Wet bioconversion rate (%) and (b) zinc accumulation (ppm) in BSFL reared on brewery spent grains fortified with ZnO-NP loaded alginate gels (0.25–5.0 % w/v) or aqueous dispersions (0.1 % w/v).

The efficacy of BSFL bioconversion and zinc enrichment on BSG was evaluated using the same ZnO–alginate gel formulations previously tested on commercial feeds. Bioconversion rates remained comparable to the control (Fig. 9a), indicating that ZnO supplementation did not hinder substrate conversion on BSG. Zinc accumulation, however, increased dose-dependently, with the 0.25% w/v gel (630 ppmZnO) yielding a modest +264 ppmZn, while the 5.0% w/v dose (12 500 ppmZnO) achieved a peak of 2865 ppm(Fig. 9b). These results demonstrate efficient Zn transfer from alginate-stabilized nanominerals and confirm that BSG effectively supports Zn biofortification without compromising bioconversion.

**Fig. 10:**
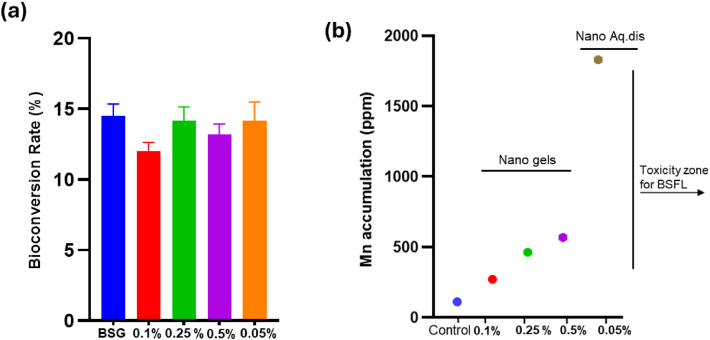
Influence of Mn_3_O_4_ nanoparticle delivery systems on the bioconversion and manganese accumulation of BSFL reared on brewery spent grains. (a) Wet bioconversion rate (%) and (b) manganese accumulation (ppm) in BSFL reared on brewery spent grains fortified with Mn_3_O_4_ loaded alginate gels (0.1–0.5 % w/v) or aqueous dispersions (0.05 % w/v).

Mn_3_O_4_ nano-formulations (0.05–0.5% w/v) were incorporated into BSG to evaluate bioconversion and Mn enrichment. Bioconversion rates were comparable to the control across most treatments (Fig. 10a), indicating that Mn_3_O_4_ supplementation did not impair feed conversion, except for a slight reduction at 0.1% w/v gel. Mn accumulation increased proportionally with Mn_3_O_4_ concentration (Fig. 10b). The dispersion delivered the highest Mn uptake (1830 ppm), exceeding both the control and gel treatments, consistent with its higher Mn load (3300 ppmper 10 kg feed) compared with gels (250–1250 ppm). Mn enrichment on BSG was also greater than that obtained using standard industry feed substrates, likely due to improved Mn solubility and absorption kinetics within the larval gut environment.

**Fig. 11:**
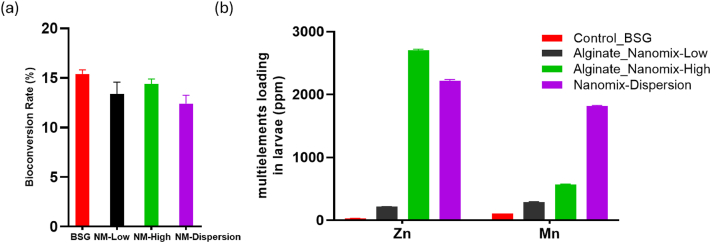
Influence of multi-element nanomineral mix (nanomix) formulations on the bioconversion and mineral accumulation of BSFL reared on brewery spent grains. (a) Wet bioconversion rate (%) and (b) zinc (Zn) and manganese (Mn) accumulation (ppm) in BSFL reared on brewery spent grains fortified with Nanomix alginate gels (low and high doses) or Nanomix aqueous dispersions.

To assess potential antagonistic interactions between co-supplied minerals, aqueous dispersions and alginate-based nanomix formulations (low and high doses of ZnO and Mn_3_O_4_, matched to concentrations previously applied to commercial feeds) were used for multi-element fortification of BSFL reared on BSG. The nanomix treatments showed enhanced Zn and Mn uptake without antagonistic effects, confirming that co-supplementation is compatible with BSFL mineral assimilation. As shown in Fig. 11a, bioconversion remained consistent across Nanomix-low, Nanomix-high, and Nanomix-dispersion treatments, all performing similarly to the unfortified BSG control. Mineral accumulation varied depending on substrate and formulation, and BSG-supported treatments yielded significantly higher Zn and Mn loading than industry-standard wheat bran and layer mash diets, particularly at ZnO (5.0% w/v) and Mn_3_O_4_ (0.05% w/v) doses (Fig. 11b). These findings demonstrate BSG's superior suitability for trace-element biofortification and highlight its strong compatibility with nanoparticle-based multi-element strategies for sustainable insect feed production.

BSFL reared on nano-fortified BSG showed clear nutritional improvements relative to the unfortified control (Table S3). ZnO alginate gels yielded the highest protein (39.3%) and ash (11.1%), reflecting efficient Zn loading and enhanced metabolic use of nitrogen. Mn_3_O_4_ gels produced the lowest fiber content (19.9% NDF DMB) and high protein (39.1%), indicating more effective substrate breakdown and conversion. Nanomix treatments delivered balanced profiles (∼39% protein, moderate fat, reduced fiber), demonstrating that multi-element supplementation supports efficient metabolism without compromising biomass quality.

Comparative analysis of proximate analysis across two substrates illustrates nano-fortification improved BSFL nutritional quality on both commercial feeds and BSG, but the effects were stronger on BSG. Alginate gels consistently produced higher protein and ash contents due to controlled mineral release and better retention, whereas dispersions yielded higher elemental uptake but slightly lower bioconversion-linked nutritional gains. On BSG, gels and dispersions generated greater increases in protein, reduced fiber more effectively, and achieved higher mineral deposition compared with wheat bran or layer mash. This enhanced performance is likely due to BSG's moisture, fiber structure, and lower baseline mineral levels, which together improve nanoparticle solubility, gut retention, and absorption. Overall, BSG + alginate gels delivered the most robust improvements, showing superior protein enrichment, stronger mineral loading, and efficient fiber reduction compared with nano-fortified commercial feeds.

### 3.4 Zinc bioavailability for poultry from BSFL reared on nanozinc fortified substrate

In this first ever provision of dietary zinc for broilers *via* BSFL reared on a nanozinc fortified substrate, trial design had to account for the possibility of low or no bioavailability of zinc from the BSFL. Therefore, in addition to a positive control of zinc provided in a commercial premix, zinc was provided at a marginal level (40 mg per kg diet) as a negative control and as a base level in the treatment assessing BSFL reared on nanozinc fortified substrate. Table 3 shows the expected response in the positive and negative controls: digestible zinc content is reduced to almost half in the 50% negative control, but the birds adapt to maintain circulating blood ionized zinc levels. In birds supplied with zinc *via* 50% premix plus nanozinc enriched insects, blood zinc level was similarly maintained at a level not significantly different from either control diet. In contrast, digestible zinc content of the diet with 50% premix plus nanozinc enriched insects, was significantly higher than both the positive control (standard premix) and the negative control. This demonstrates BSFL reared on nanozinc fortified substrate provided a form of zinc with significantly higher digestibility than standard premixes to poultry.

**Table 3:** Digestible zinc content and serum zinc level of broilers fed with zinc oxide within a commercially source vitamin-mineral premix or nanozinc *via* black soldier fly larvae reared on enriched substrate.

Zinc source	Blood serum Zn (ppm)	Digestible Zn content (g/kg diet)
Premix at 50% commercial standard	25.1	3.6c
50% Premix plus nanozinc enriched insects	21.6	6.5a
Premix at commercial standard level	25.7	6.4b

SEM	2.75	3.5 x10^−5^
P	0.589	<0.001

## 4 Economics and sustainability of nanomineral delivery *via* BSFL

Evaluation of delivery routes for Zn-based nanominerals demonstrated clear economic and operational distinctions between nanoparticle dispersions and alginate-based gels. Water-based dispersions represented the lowest-cost option, at approximately £0.10 per kg, whereas alginate gels were substantially more expensive at ∼£0.70 per kg and added ∼£0.175 per 10 kg substrate tray. Despite higher cost, alginate gels provided a more concentrated and controlled delivery mechanism, reducing the likelihood of metallic shock and improving mineral stability during feeding. These contrasting delivery efficiencies indicate that cost minimization favors water dispersions, while controlled nutrient release and operational precision favor alginate systems.

Sustainability modelling further quantified the fate of supplemented Zn within the bioconversion system. Across 10 kg substrate trials yielding ∼1.5 kg larvae, Zn-fortified treatments increased larval Zn content by ∼0.180 g relative to unfortified controls, representing a 28.8% mineral conversion efficiency from the 0.625 g nano-Zn applied. Although the remaining Zn (∼71.2%) persisted in the frass, this fraction constituted a valuable co-product rather than a loss, as Zn-enriched frass is commercially utilized as a nutrient-enhanced organic fertilizer. This dual-stream recovery pathway, biofortified larvae and mineral-rich frass, substantially improves material efficiency relative to conventional supplementation approaches, where non-bioavailable minerals are often wasted.

Economic comparison against standard feed-grade Zn further highlighted the commercial potential of nano-enabled supplementation. Although nano-Zn is a higher-value material, its effective inclusion rate is lower (50 g t^−1^ feed) compared with standard ZnO (80 g t^−1^), resulting in a lower overall cost: £0.825 t^-1^ for nano-Zn versus £1.155 t^-1^ for standard ZnO. When adjusted for bioaccumulation efficiency and the value of Zn-enriched frass, nano-Zn delivers both economic savings and higher resource utilisation efficiency. Additionally, the resulting BSFL biomass provides functional advantages, such as on-demand mineral delivery and flexible nutrient profiles, which further strengthen commercial viability.

Collectively, these economic and sustainability outcomes demonstrate that nanoparticle-based fortification is not only biologically effective but also materially efficient and cost-advantageous, particularly when both larval biomass and frass are integrated into circular-economy feed and fertilizer pathways.

## 5 Conclusions

This study presents a commercially viable and scalable strategy for sustainable insect protein meal production by synthesizing and applying ZnO, Mn_3_O_4_, CuO, and Se nanoparticles in BSFL bioconversion systems. Using a spinning disc reactor, highly bioavailable mineral nanoparticles were formulated into alginate gels and aqueous dispersions for controlled delivery *via* two distinct substrates: brewery spent grains (BSG) and wheat bran with layer mash (WBLM). BSFL reared on BSG achieved effective multi-element fortification while valorizing a high-moisture agro-industrial waste, whereas WBLM served as a nutritionally adequate matrix for mineral enhancement. Across both substrates, nanoparticle supplementation significantly improved BSFL protein and fat content while reducing fiber levels, confirming enhanced digestibility and nutrient conversion. Across both systems, nanoparticle supplementation resulted in safe and efficient uptake of all target elements, with no observed toxicity or antagonistic interactions, even under multi-element nanomix conditions, indicating safe and synergistic uptake. This integrated approach supports the production of low-cost, low carbon-emission mineral-enriched insect protein meal, aligning with circular bioeconomy principles and regulatory frameworks for feed safety. Furthermore, the ability to generate larvae with enhanced nutritional value offers strong commercial relevance and product strategy insights for BSFL producers, enabling greater integration into poultry feed markets and advancing the role of insect-based ingredients in sustainable animal nutrition. Future work will focus on long-term feeding trials in livestock and developing nanofortified BSFL frass as a nutrient-dense, sustainable growing medium to support circular nutrient recovery and advance sustainable agricultural practices.

## Funding sources

This work was supported by Innovate UK and Biotechnology and Biological Sciences Research Council (Project number 10073997); *Nutritional enhancement of waste and Black Soldier Fly Meal (BSFLM) to establish the UK as a global leader in BSFLM industry economics),* under the *Novel low-emission food production systems: Industrial Research programme*.

## CRediT authorship contribution statement

**Subbareddy Mekapothula:** Data curation, Formal analysis, Investigation, Visualization, Writing – original draft. **Bridget Ristow:** Formal analysis, Methodology, Writing – original draft. **David Stanford-Beale:** Data curation, Formal analysis, Investigation, Methodology. **Ashraf Alkhtib:** Formal analysis, Methodology, Validation. **Dawn Scholey:** Funding acquisition, Methodology, Validation. **Thomas Stringer:** Conceptualization, Funding acquisition, Project administration, Supervision. **Emily J. Burton:** Funding acquisition, Methodology, Supervision, Validation, Writing – review & editing. **Gareth W.V. Cave:** Conceptualization, Funding acquisition, Project administration, Supervision, Validation, Writing – review & editing.

## Declaration of competing interest

The authors declare that they have no known competing financial interests or personal relationships that could have appeared to influence the work reported in this paper.

## Data Availability

Data will be made available on request.

## Supporting Information

Details of Synthesis of nanominerals, characterisation of nanominerals, nanomineral loaded alginate gels and their characterisation, preparation of commercial standard and brewery spent grains feed substrates for BSFL rearing, nanomineral fortified BSFL nutritional and proximate analysis, nanozinc fortified BSFL loaded poultry diet preparation and poultry feeding trial to determine digestible zinc content in broilers.

## Appendix A Supplementary data

Multimedia Component1
