# Supplementary material for: Nutritional enrichment of black soldier fly larvae towards a sustainable protein food supplement for livestock and poultry production
Source: Clean Food Syst. 2026 Jun;3:None. doi: 10.1016/j.clfs.2026.100042 (PMC13288454; doi:10.1016/j.clfs.2026.100042)
Supplement: Multimedia Component1 [file mmc1.docx]

Supplementary Information

Nutritional Enrichment of Black Soldier Fly Larvae Towards Sustainable Protein Food Supplement For Livestock and Poultry Production

*Subbareddy Mekapothula,^1^ Bridget Ristow,^2^ David Stanford-Beale,^2^ Ashraf Alkhtib^3^, Dawn Scholey^3^, Thomas Stringer,^2^ Emily Burton,^3^ and Gareth W. V. Cave*^1^*

Authors

^1^Subbareddy Mekapothula - School of Science and Technology, Nottingham Trent University, Nottingham, NG11 8NS, United Kingdom

^2^Bridget Ristow - FlyBox, The Royal Institution, 21 Albemarle Street, London, W1S 4BS, United Kingdom

^2^David Stanford-Beale - FlyBox, The Royal Institution, 21 Albemarle Street, London, W1S 4BS, United Kingdom

^2^Thomas Stringer - FlyBox, The Royal Institution, 21 Albemarle Street, London, W1S 4BS, United Kingdom

^3^Ashraf Alkhtib - Nottingham Trent University Poultry Research Unit, Southwell, NG25 0QF, United Kingdom

^3^Dawn Scholey - Nottingham Trent University Poultry Research Unit, Southwell, NG25 0QF, United Kingdom

^3^Emily Burton - Nottingham Trent University Poultry Research Unit, Southwell, NG25 0QF, United Kingdom

Corresponding Author

^1^*Gareth W. V. Cave – School of Science and Technology, Nottingham Trent University, Nottingham, NG11 8NS, United Kingdom; Email: Gareth.Cave@ntu.ac.uk

1. **Experimental Section**

**Reagents and Materials**

All chemicals and solvents were purchased as reagent grade or mass spectrometric grade and used without any further purification. Zinc Chloride anhydrous, manganese nitrate, copper(II) chloride anhydrous, selenium tetrachloride with poly(4-sodiumstyrenesulphonate), L-(+)-Ascorbic acid, sodium hydroxide (Glentham Life Sciences, Corsham, UK) was used to synthesize zinc, manganese, copper and selenium nanoparticles using spinning disc reactor. Sodium alginate (special ingredients, UK) used to prepare alginate gels. Black Soldier Fly Larvae were received from FlyBox (Chesham, UK), Layers mash (Mole Valley Farmers, UK) and wheat bran (Target Feeds, UK), Brewery Spent Grains (Sambrook’s Distillery)were used as feed substrates. HNO_3_ (67-70%, Trace Metal™, for Trace Metal analysis, Fisher Scientific) and H_2_O_2_ (≥30%, for trace analysis, Merck). Moisture analyzer used to determine the moisture content of feed substrates and BSFL and blue insect breeding trays (Beekenkamp, 60 x 40 x 14.5) were used to breed BSFL. Wire tray desk organizer (Zuvo mesh 4 tier letter) to separate the 4 DOLs from the frass.

1. **Elemental analysis of BSFL life cycle and BSFL feed substrates**

Wheat bran, layers mash, brewery spent grains, Black Soldier Fly eggs, 4DOLs, 12DOLs, pre-pupae, pupae, adults, frass were finely grinded separately using blender after freeze drying. Subsequently, samples (0.25 g, 3 replicates) were loaded into high pressure microwave vessel (100 mL) containing ultrapure nitric acid (9 mL) and H_2_O_2_ (1 mL) for microwave digestion at 1800 (w) power and temperature T1 at 200°C and T2 at 140°C in 10 min and steady for 30 mins before it cool down to room temperature in 30 min . Furthermore, the digested solutions were filtered into volumetric flasks (100 mL, ) through filter papers (Whatman 451) and continue to rinse the conical flasks with ultra-pure water and filter the solution until 2-3 times. Bring the volumetric to volume with ultra-pure water. Certipur® ICP Single-Element standards (Zn, Mn, Cu, Se, Mg, Co, Fe, Mo and Si) were used to create a calibration curve to determine the levels of elements in each sample. The calibration range was defined as 1,000 ppb to 50 ppb. Microwave reactor (Analytix, Ethos UP, SR-15) and Inductive-Coupled Mass Spectrometry (ICP-MS, PerkinElmer NexION 1000, Waltham, MA, USA) were used for elemental analysis of feed substrates, BSFL life cycle, nanoparticles loaded BSFL.

1. **Synthesis and characterization of nanoparticles using spinning disc reactor**

In brief, Metal oxide nanoparticles of ZnO, CuO, Mn_3_O_4_, and Se were synthesized using a spinning disc reactor (SDR) to achieve controlled particle formation and size distribution. Aqueous solutions of metal salts and sodium hydroxide were prepared at desired concentrations depending on the target metal oxide. These solutions were simultaneously pumped (60 mL/min) onto the center of a rotating disc (15 cm diameter, 1500 rpm), where rapid mixing and reaction occurred on the disc surface, leading to spontaneous nanoparticle formation.

For ZnO synthesis, zinc chloride anhydrous (1 M) and sodium hydroxide (3 M) were used to pump at 60 mL/min with rotating disc set to 1200 rpm. CuO nanoparticles were synthesized using copper(II) chloride (1 M) and sodium hydroxide (2 M) at 90 °C at 60 mL/min with rotating disc set to 1100 rpm. Mn_3_O_4_ nanoparticles were produced from manganese nitrate (1 M) and sodium hydroxide (2 M) at 60 mL/min with rotating disc set to 1200 rpm. Se nanoparticles were synthesized using selenium tetrachloride (0.25 M) in the presence of poly(sodium 4-styrenesulfonate) (PSS, 2% w/v) and ascorbic acid (0.63 M) as a reducing agent at 60 mL/min with rotating disc set to 1000 rpm. The resulting nanoparticle suspensions were collected and filtered under gravity using a sintered glass funnel (porosity grade 3). The filter cakes were washed with deionized water (3 × 250 mL) and dried in a hot air oven at 120 °C for approximately 3 hours prior to characterization.

Morphological analysis of the nanoparticles was performed using scanning electron microscopy (SEM, JEOL JSM-7100F, Tokyo, Japan) after sputter coating (Quorum Q150R, East Sussex, UK). Particle size and shape were further examined using transmission electron microscopy (TEM, JEM-2100 Plus, JEOL, Tokyo, Japan) with carbon film-coated copper grids (Agar Scientific Ltd., Stansted, UK). Additional physicochemical characterization included powder X-ray diffraction (XRD, Rigaku Co., Ltd., Tokyo, Japan), thermogravimetric analysis (TGA 4000, PerkinElmer, Beaconsfield, UK), and zeta potential measurements using a Zetasizer Nano ZS (Malvern Panalytical Ltd., Malvern, UK). ImageJ software (Version 1.54i, 3 March 2024, USA) was used for image-based particle size analysis.


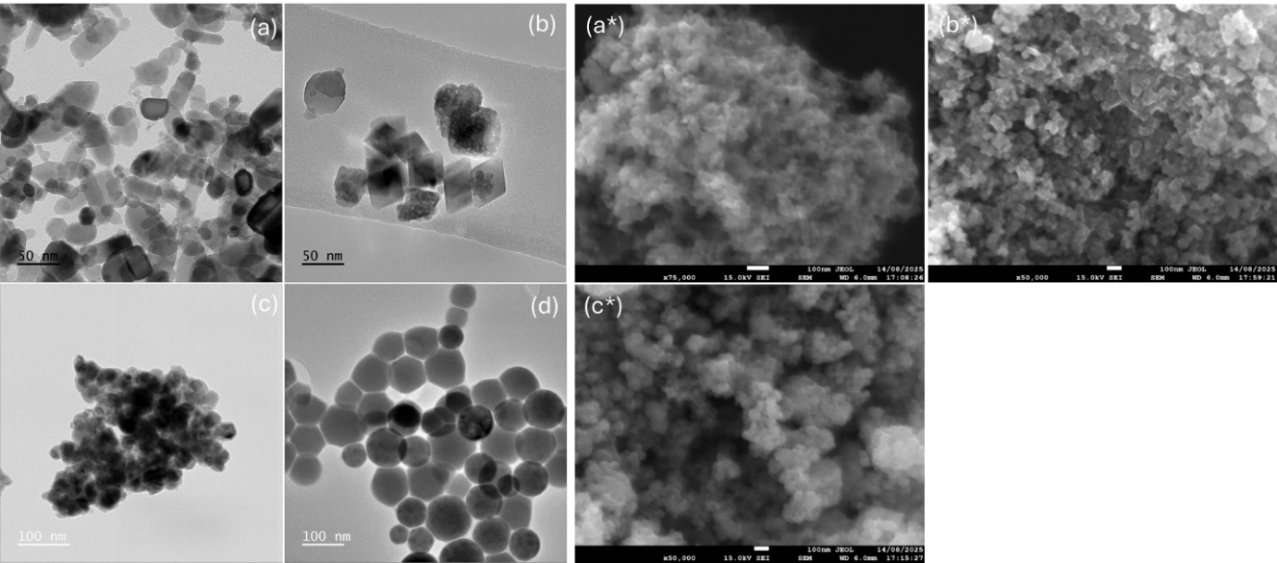


Figure S1. TEM and SEM images of Zinc Oxide nanoparticles (a and a*), Manganese Oxide nanoparticles (b and b*), Copper Oxide nanoparticles (c and c*), and Selenium nanoparticles (d).


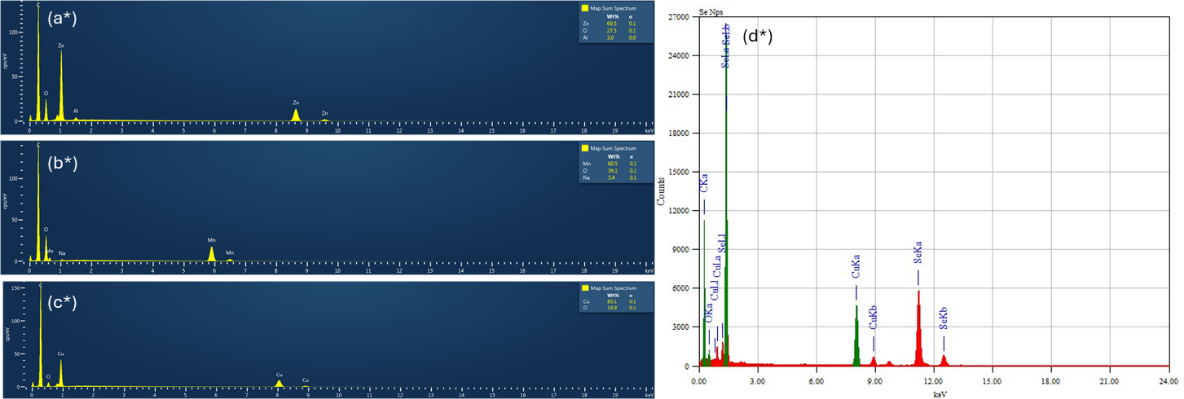


Figure S2. Energy dispersive X-ray spectroscopy (EDS) of ZnO, Mn_3_O_4_, CuO and Se nanoparticles.


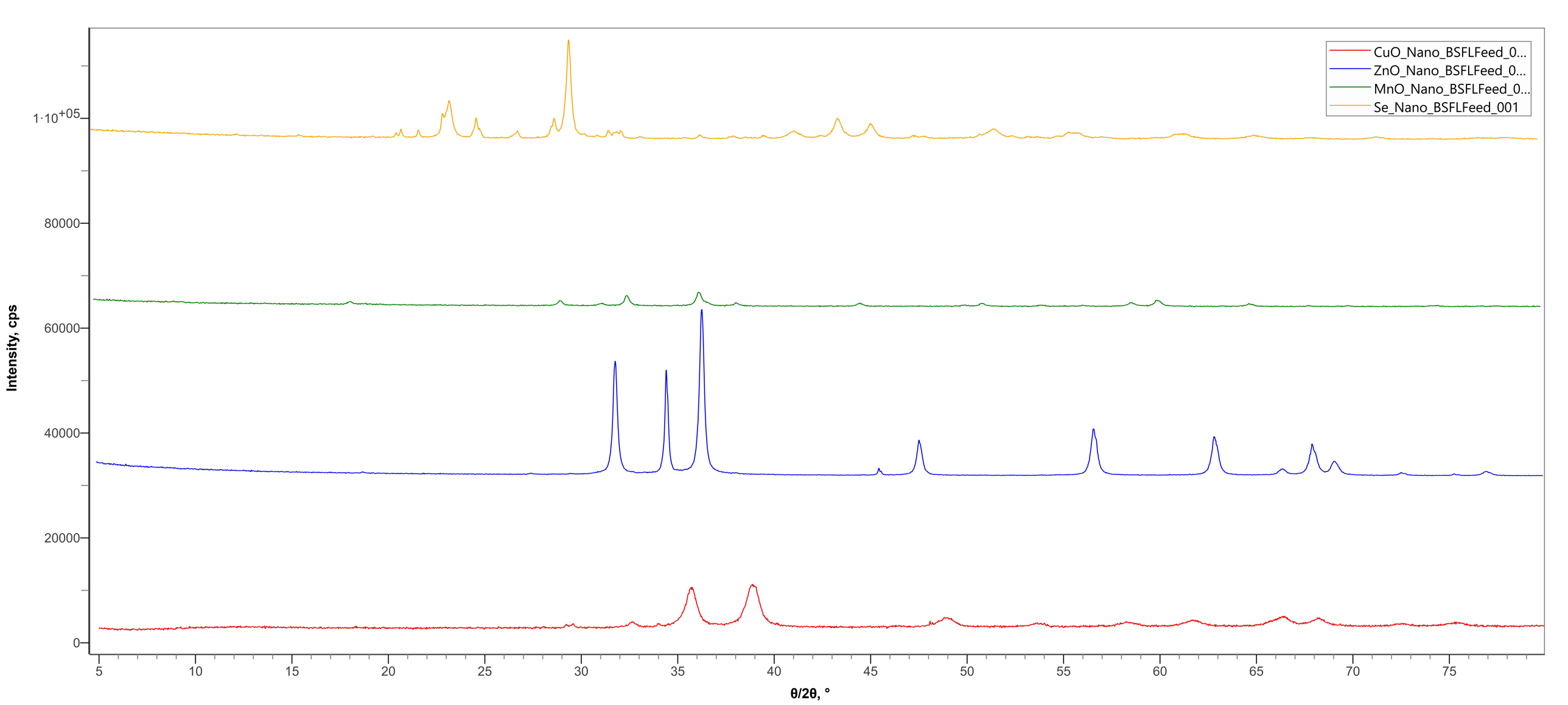


Figure S3. Powdered X-Ray Diffraction of CuO, ZnO, Mn_3_O_4_, and Se nanoparticles.


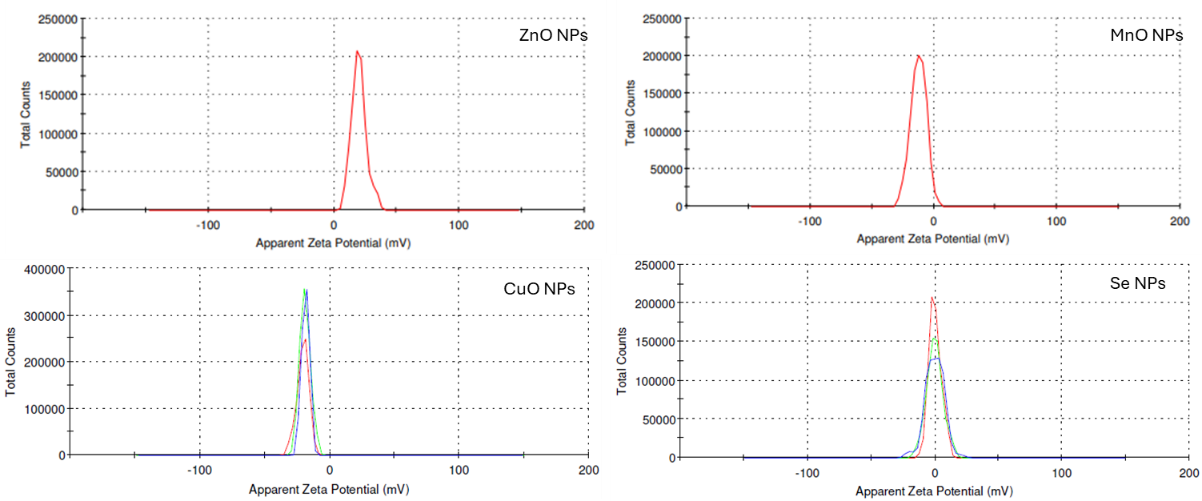


Figure S4. Zeta potential analysis of ZnO, Mn_3_O_4_, CuO, and Se nanoparticles


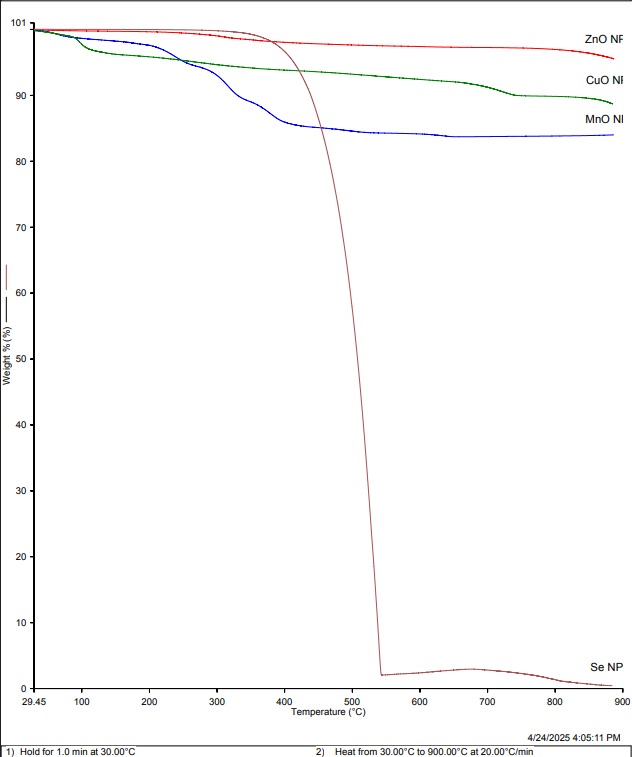


Figure S5. Thermogravimetric analysis of ZnO, CuO, Mn_3_O_4_, and Se nanoparticle

**Synthesis and characterization of nanoparticle loaded sodium alginate gels and nano dispersions**

Metal oxide nanoparticles (ZnO, Mn_3_O_4_, CuO, and Se) were formulated into two types of delivery systems, alginate-based gels and aqueous dispersions, for the fortification of black soldier fly larvae (BSFL) feed substrates. For gel-based formulations, nanoparticles were first dispersed in deionized water (50 mL) and then incorporated into a sodium alginate solution (200 mL, 4% w/v) under continuous stirring to form nanoparticle-loaded alginate gels. The gels were prepared using the following concentrations: ZnO at 0.25%, 0.5%, 1.0%, 2.5%, and 5.0% (w/v); CuO and Mn_3_O_4_ at 0.1%, 0.25%, and 0.5% (w/v); and Se at 0.06%, 0.09%, 0.12%, and 0.18% (w/v). These gels were characterized using inductively coupled plasma mass spectrometry (ICP-MS) to quantify Zn, Cu, Mn, and Se content, and scanning electron microscopy with energy-dispersive X-ray spectroscopy (SEM-EDS) to assess elemental distribution and gel morphology. In parallel, aqueous nanoparticle dispersions were prepared using ZnO (0.1% w/v), Mn_3_O_4_ (0.05% w/v), CuO (0.1% w/v), and Se (0.09% w/v).


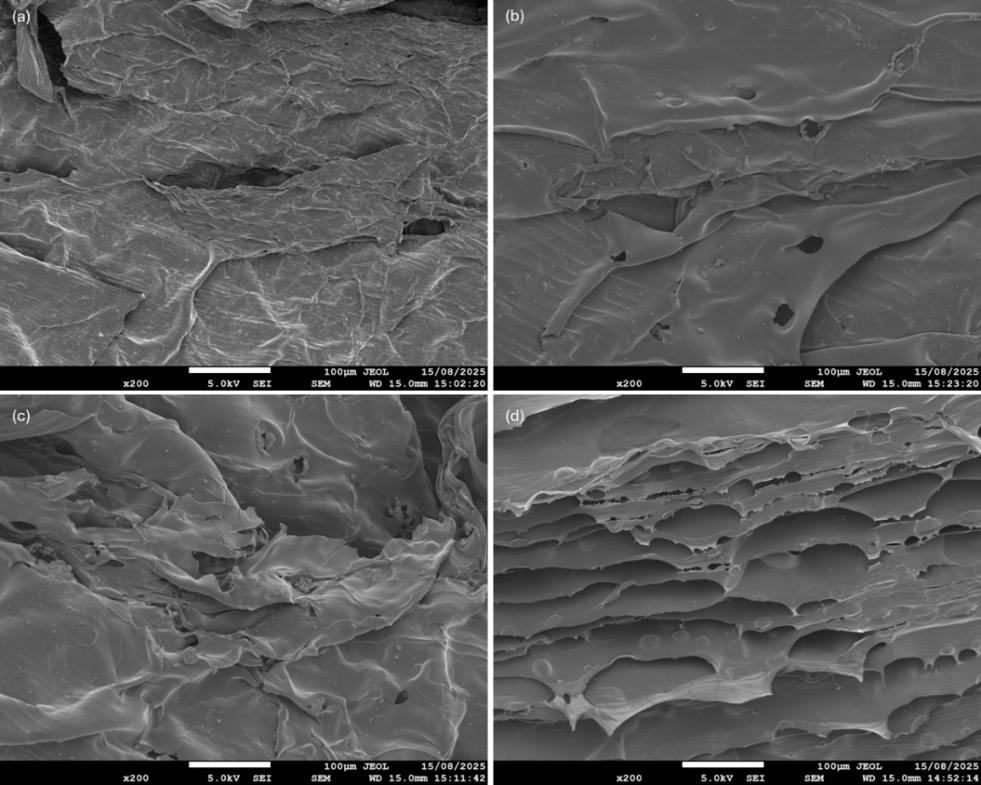


Figure S6. Scanning electron microscopic images of alginate gels loaded with ZnO NPs (a), Mn_3_O_4_ NPs (b), CuO NPs (c), and Se NPs (d).

**
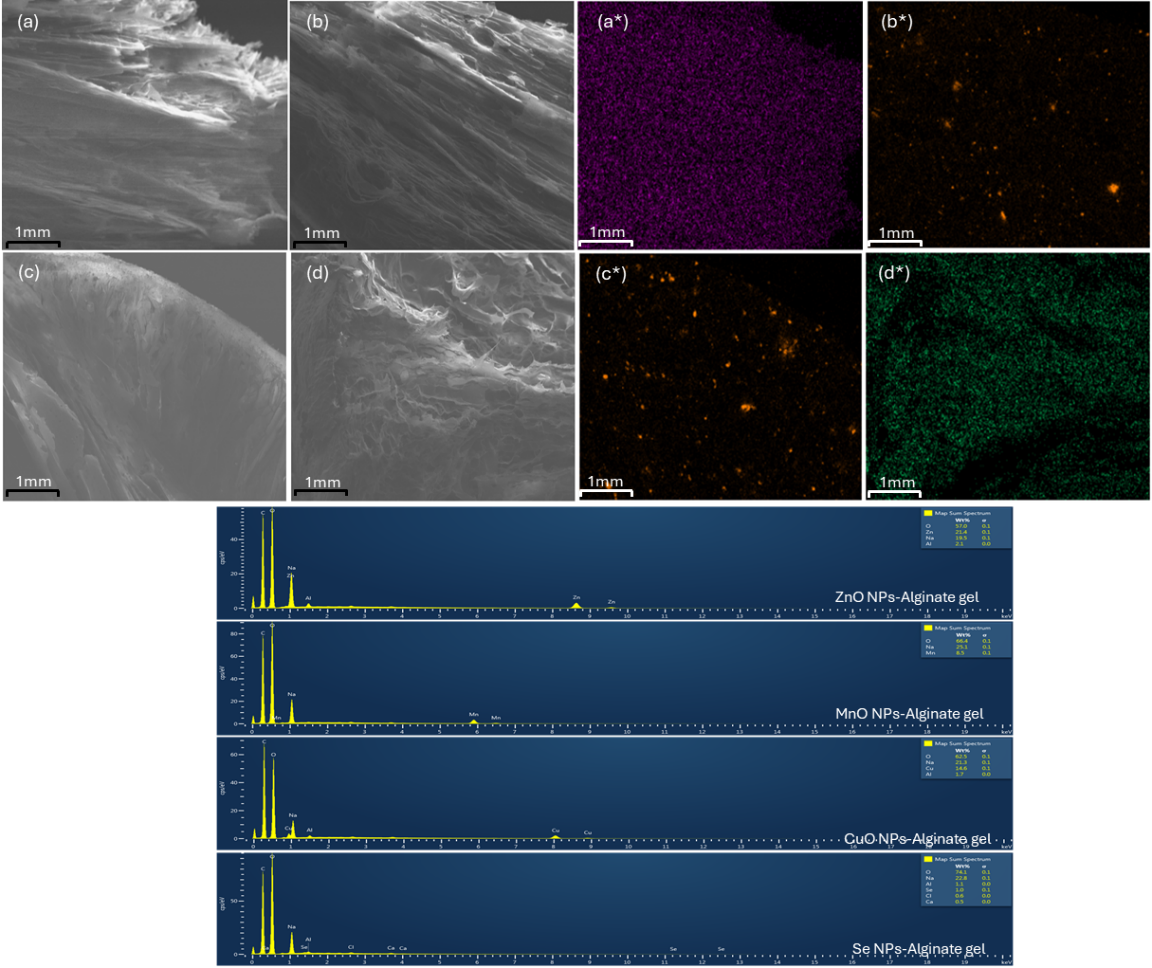
**

Figure S7. SEM imaging and energy-dispersive X-ray spectroscopy (EDS) characterization of ZnO (a, a*), Mn_3_O_4_ (b, b*), CuO (b, b*), and Se (d,d*) nanoparticle loaded sodium alginate gels.

Table S1. ICP-MS elemental analysis of ZnO, Mn_3_O_4_, CuO, and Se nanoparticle loaded sodium alginate gel

| ICP-MS ZnO loaded sodium alginate gels | | |
| --- | --- | --- |
| Treatment (w/v) | ZnO Concentration in gel (g) | Identified ZnO Concentration (g) |
| Alginate_ZnO_0.25% | 0.625 | 0.63 ± 0.01 |
| Alginate_ZnO_0.5% | 1.25 | 1.25 ± 0.01 |
| Alginate_ZnO_1.0% | 2.5 | 2.49 ± 0.02 |
| Alginate_ZnO_2.5% | 6.25 | 6.25 ± 0.01 |
| Alginate_ZnO_5.0% | 12.5 | 12.55 ± 0.02 |
| Dispersion ZnO_0.1% | 6.6 | 6.59 ± 0.02 |
| ICP-MS Mn_3_O_4_ loaded sodium alginate gels | | |
| Treatment (w/v) | Mn_3_O_4_ Concentration in gel (g) | Identified Mn_3_O_4_Concentration (g) |
| Alginate_ Mn_3_O_4__0.1% | 0.25 | 0.25 ± 0.01 |
| Alginate_ Mn_3_O_4__0.5% | 0.625 | 0.63 ± 0.01 |
| Alginate_ Mn_3_O_4__1.0% | 1.25 | 1.25 ± 0.01 |
| Disperison_ Mn_3_O_4__0.05% | 3.3 | 3.29 ± 0.01 |
| ICP-MS CuO loaded sodium alginate gels | | |
| Treatment (w/v) | CuO Concentration (g) | Identified CuO Concentration (g) |
| Alginate_CuO_0.1% | 0.25 | 0.24 ± 0.01 |
| Alginate_CuO_0.5% | 0.625 | 0.625 ± 0.02 |
| Alginate_CuO_1.0% | 1.25 | 1.25 ± 0.01 |
| Disperison_CuO_0.1% | 6.6 | 3.29 ± 0.01 |
| ICP-MS Se loaded sodium alginate gels | | |
| Treatment (w/v) | Se Concentration in gel (g) | Identified Se Concentration (g) |
| Alginate_Se_0.06% | 0.15 | 0.15 ± 0.01 |
| Alginate_Se_0.09% | 0.225 | 0.23 ± 0.01 |
| Alginate_Se_0.12% | 0.3 | 0.31 ± 0.02 |
| Alginate_Se_0.18% | 0.45 | 0.45 ± 0.01 |
| Dispersion Se_0.09% | 0.225 | - 1. 0.02 |

1. **BSFL rearing on commercial feed substrates and brewery spent grains waste**
2. Substrate Preparation and Environmental Control: Experimental tubs were labeled with the project ID, treatment designation, replicate number (1-3), date of setting, and anticipated harvest date. Prior to trial setup, the climate-controlled environment was stabilized at 30 °C and 60–65% relative humidity (RH), CO_2_ levels 2000 ppm according to FlyBox standard operating conditions. Air filter pressure, extractor fans were monitored regularly and recorded/stored in the Entopod system.

Commercial industry feedstock was prepared by using layers mash (2.4 kg), wheat bran (1.0 kg) and Water (6.6 L) using mechanical mixing, transferred to labeled experimental blue trays to a uniform depth (5 ± 1 cm) and stored in the controlled environment chamber stored for 24 hours prior to larval addition. Moisture content was adjusted to 70 ± 2% and determined using a moisture content analyzer.

The brewery spent grain (BSG) diet was formulated using 80% wheat spent grains, 10% fine maize (added to provide energy), and 10% water, sourced from Sambrook’s Brewery without any preprocessing, milling, or particle size reduction. For each 10 kg batch, 8 kg of spent grains, 1 kg of fine maize, and 1 kg of wheat bran were mixed with water to achieve the target moisture content (70 ± 2%) and loaded into trays at a uniform depth of approximately 5 cm.

An additional 500 g of each formulation was collected in labeled ziplock bags and frozen for downstream analytical characterization.

ZnO, CuO, Mn_3_O_4_ and Se nanoparticle alginate gels and dispersions at various concentrations (% w/v) were loaded into each substrate at a inclusion rate of 2.5% (w/w) to study the growth performance, nutritional accumulation and bioconversion rate.

1. Larval Inoculation and Growth Conditions: Four-day-old larvae were separated from nursery residue using a sieve system that allowed active larvae to self-sort into collection trays. Larvae dosing was performed to determine the wight of larvae equal to 7500 larvae dosing per treatment. The total larval mass for each replicate was recorded to calculate average individual larval weight. Equivalent larval masses were transferred to each treatment tray, maintaining consistent stocking density across replicates. Trays were stacked on each other while leaving bottom two trays and top one tray to allow consistent airflow, to incubate in the controlled environment (30 °C, 60–65% RH) for an 8-day growth period. Throughout the trial, environmental conditions were maintained within set thresholds to ensure consistency in larval development and substrate degradation.
2. Harvest and Biomass Separation: At the end of the grow-out period, larvae were separated from the residual frass using a sieve system and immediately weighed to determine total larval yield for each replicate. After weighing, the larvae (500 g) were immobilized using dry ice to prevent post-harvest movement or escape and subsequently transferred to the laboratory for nutritional and proximate analysis. A representative subsample of 30 larvae was collected from each replicate to determine individual larval weight. Frass was also collected in labelled containers, freeze-dried, and prepared for moisture content analysis.
   1. **Proximate and nutritional analysis of feed substrates and nano fortified BSFL**

Feed substrates, BSFL, nanofortified BSFL were subjected to determine the proximate analysis, Ash value, fat content, nitrogen and crude protein content, and fiber content (dry matter). Ash value was determined by combusting all organic (carbon based) material within the sample to measure the mineral ash remains. Fat content was determined *via* Soxtherm extraction using petroleum ether. Nitrogen content of the feed substrates, BSFL, nanofortified BSFL were determined using a combustion analyser (Dumatherm N Pro, Gerhardt Analytical Systems, Königswinter, Germany) then multiplied by 6.25 to derive crude protein content. Extractable fat content, dry matter and was analysed Fiber Analyzer (ANKOM200). The nanofortified BSFL was subjected to microwave digestion to determine elemental concentration.

Table S2. Proximate analysis of BSFL industry feed substrate and BSFL fortified with nano (ZnO, Mn_3_O_4,_CuO, and Se) via alginate gels and aqueous dispersions on wheat bran and layers mash.

| **Treatment Type** | **% Ash** | **% Fat** | **% NDF DMB** | **% Protein** |
| --- | --- | --- | --- | --- |
| Wheat bran | 10.97 ± 0.09 | 3.53 ± 0.50 | 34.95 ± 0.52 | 15.96 ± 0.36 |
| Layers mash | 3.43 ± 0.13 | 4.99 ± 0.05 | 16.41 ± 0.54 | 18.21 ± 0.22 |
| BSFL-control | 10.27 ± 0.00 | 24.97 ± 0.69 | 7.89 ± 0.36 | 38.31 ± 0.83 |
| Alginate_ZnO_0.25% | 10.28 ± 0.00 | 22.40 ± 0.46 | 8.60 ± 0.27 | 37.78 ± 0.07 |
| Alginate_ZnO_0.5% | 10.79 ± 0.50 | 21.79 ± 0.09 | 10.26 ± 0.53 | 38.95 ± 0.10 |
| Alginate_ZnO_1.0% | 10.80 ± 0.51 | 22.96 ± 0.07 | 9.54 ± 0.30 | 37.87 ± 0.40 |
| Alginate_ZnO_2.5% | 11.31 ± 0.00 | 22.52 ± 0.03 | 8.25 ± 0.25 | 36.92 ± 0.75 |
| Alginate_ZnO_5.0% | 11.82 ± 0.50 | 24.60 ± 0.17 | 11.21 ± 2.42 | 39.50 ± 0.01 |
| Dispersion_ZnO_0.1% | 10.81 ± 0.50 | 22.52 ± 0.67 | 10.89 ± 1.61 | 38.41 ± 0.66 |
| Alginate_ Mn3O4_0.1% | 11.93 ± 0.07 | 22.29 ± 0.26 | 11.59 ± 0.42 | 37.71 ± 0.44 |
| Alginate_ Mn_3_O_4__0.25% | 11.63 ± 0.07 | 22.05 ± 0.07 | 9.33 ± 0.40 | 37.54 ± 0.53 |
| Alginate_ _Mn3O4__0.5% | 11.95 ± 0.08 | 19.91 ± 2.39 | 9.47 ± 0.59 | 38.30 ± 0.49 |
| Disperison_ Mn_3_O_4__0.05% | 12.00 ± 0.12 | 18.70 ± 0.04 | 9.97 ± 0.14 | 38.36 ± 0.57 |
| Alginate_CuO_0.1% | 11.85 ± 0.25 | 23.26 ± 1.38 | 17.57 ± 2.33 | 37.14 ± 0.44 |
| Alginate_CuO_0.25% | 11.75 ± 0.05 | 23.10 ± 0.41 | 16.06 ± 0.52 | 36.92 ± 0.69 |
| Alginate_CuO_0.50% | 11.35 ± 0.05 | 23.35 ± 0.07 | 19.89 ± 2.48 | 38.40 ± 0.62 |
| Disperison_CuO_0.1% | 9.40 ± 0.00 | 28.36 ± 0.14 | 24.65 ± 2.94 | 37.65 ± 0.18 |
| Alginate_Se_0.06% | 12.12 ± 0.21 | 22.07 ± 0.07 | 10.87 ± 1.10 | 37.96 ± 0.39 |
| Alginate_Se_0.09% | 12.37 ± 0.03 | 30.34 ± 0.35 | 9.10 ± 0.48 | 38.50 ± 0.33 |
| Alginate_Se_0.12% | 12.33 ± 0.03 | 29.93 ± 1.94 | 9.27 ± 0.40 | 38.33 ± 0.54 |
| Alginate_Se_0.18% | 11.77 ± 0.07 | 25.65 ± 2.51 | 9.17 ± 0.10 | 38.31 ± 0.46 |
| Dispersion_Se_0.09% | 11.40 ± 0.14 | 24.88 ± 0.41 | 9.50 ± 0.08 | 37.66 ± 0.53 |
| Alginate_nanomix_low | 12.23 ± 0.09 | 19.80 ± 0.20 | 17.39 ± 0.46 | 37.16 ± 0.26 |
| Alginate_nanomix_high | 11.53 ± 0.18 | 25.18 ± 0.42 | 19.76 ± 0.69 | 37.93 ± 0.34 |
| Nanomix_dispersion | 10.20 ± 0.66 | 25.60 ± 1.67 | 16.55 ± 0.90 | 38.24 ± 0.50 |

Table S3 Proximate analysis of brewery spent grains and BSFL fortified with nano (ZnO, Mn_3_O_4_, CuO, and Se) via alginate gels and aqueous dispersions on BSG.

| **Treatment Type** | **% Ash** | **% Fat** | **% NDF DMB** | **% Protein** |
| --- | --- | --- | --- | --- |
| Brewery spent grains | 3.43 ± 0.13 | 4.99 ± 0.05 | 52.37 ± 0.75 | 26.30 ± 0.26 |
| Alginate_ZnO_0.25% | 11.03 ± 0.12 | 14.04 ± 0.06 | 27.96 ± 1.10 | 39.00 ± 0.62 |
| Alginate_ZnO_0.5% | 11.13 ± 0.12 | 11.22 ± 2.39 | 25.54 ± 0.31 | 39.12 ± 0.27 |
| Alginate_ZnO_1.0% | 11.37 ± 0.03 | 18.13 ± 1.72 | 22.08 ± 0.45 | 40.78 ± 1.35 |
| Alginate_ZnO_2.5% | 9.90 ± 0.20 | 14.03 ± 1.22 | 20.14 ± 0.26 | 39.03 ± 0.72 |
| Alginate_ZnO_5.0% | 11.80 ± 0.15 | 12.79 ± 0.14 | 29.17 ± 0.28 | 38.61 ± 0.56 |
| Disperison ZnO_0.1% | 9.93 ± 0.19 | 14.93 ± 0.02 | 25.45 ± 1.14 | 38.65 ± 0.37 |
| Alginate_ Mn_3_O_4__0.1% | 9.72 ± 0.12 | 15.70 ± 0.92 | 18.38 ± 0.58 | 39.26 ± 0.41 |
| Alginate_ Mn_3_O_4__0.25% | 9.67 ± 0.15 | 12.80 ± 0.10 | 17.49 ± 0.25 | 38.87 ± 0.39 |
| Alginate_ Mn_3_O_4__0.5% | 10.15 ± 0.03 | 14.38 ± 0.25 | 23.96 ± 0.68 | 38.86 ± 0.36 |
| Disperison_ZnO_0.05% | 9.98 ± 0.12 | 16.97 ± 1.13 | 21.43 ± 1.31 | 38.72 ± 0.48 |
| Alginate_nanomix_low | 8.73 ± 0.55 | 12.91 ± 0.93 | 18.56 ± 0.36 | 39.02 ± 0.36 |
| Alginate_nanomix_high | 12.13 ± 0.58 | 12.23 ± 1.34 | 24.22 ± 0.81 | 38.58 ± 0.30 |
| Nanomix_dispersion | 11.07 ± 0.15 | 15.10 ± 2.07 | 21.29 ± 0.46 | - 1. 0.38 |


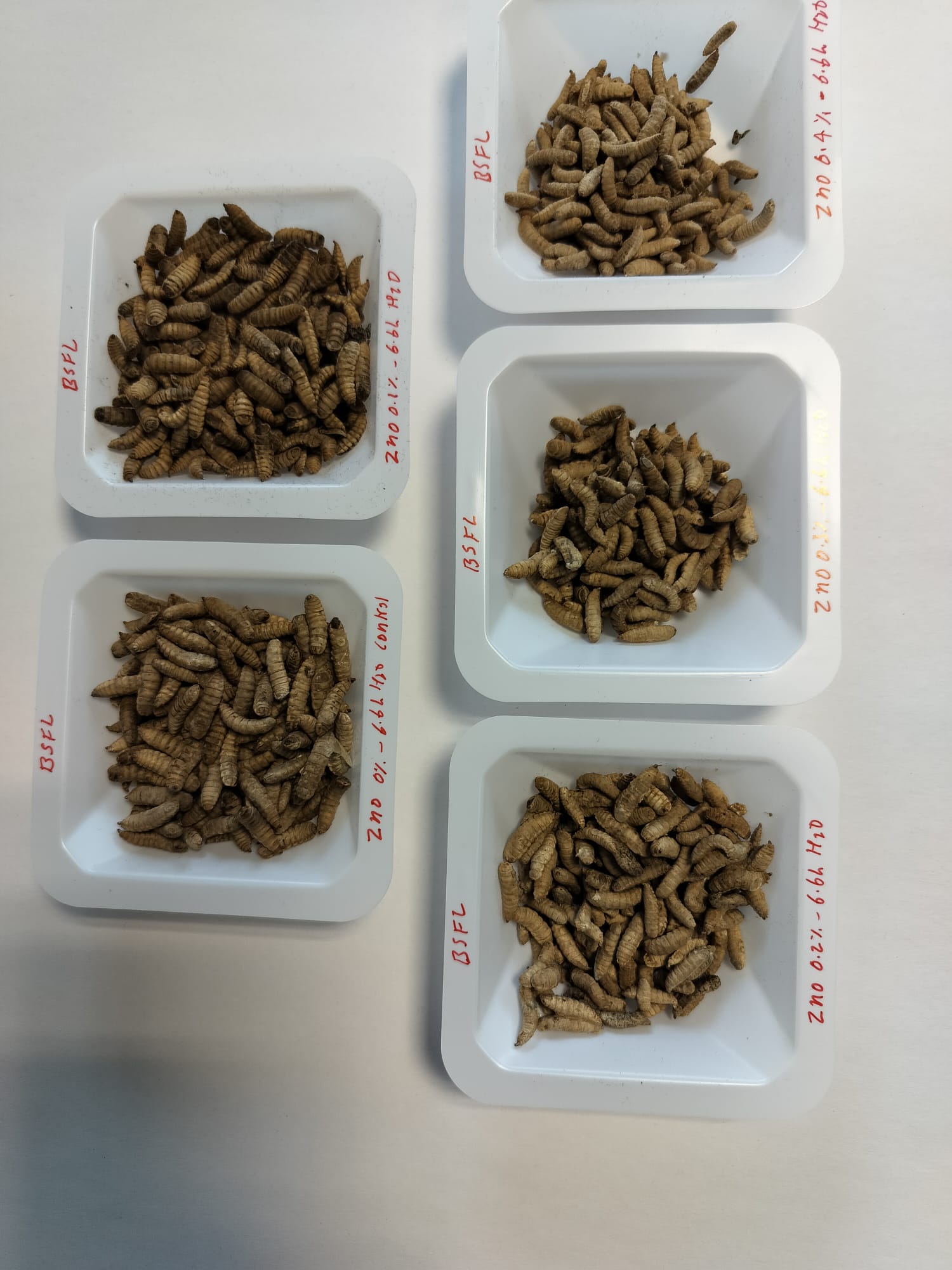


Figure S4: BSFL rearing on ZnO NPs dispersion fortified BSFL industry feed substrate (wheat bran and layers mash).

1. Study title: Digestibility of zinc provided by insects to broilers

Poultry Data Report

# 5.1 Summary

A trial was designed to compare the zinc digestibility of a diet containing 50/50 insect larvae and premix in comparison to a 50% premix diet and a 100% premix diet. This study also recorded the effect of insect larvae on bodyweight gain in broilers from d29 to d32. Birds were raised on commercial chick starter diet from day of hatch until d29 and then transferred onto one of the 3 diets with either 50% premix, 50% premix plus insects or 100% premix.

Each diet also contained titanium dioxide at 5g/kg as an inert marker for digestibility measures, with added vit/min mix and insect larvae (See table 2). Birds were culled on d32. Ileal digesta (defined as the intestinal portion between Meckel’s Diverticulum and the ileal-caecal-colonic junction) was collected post mortem on d32 and pooled into one pot (2 birds per set and 2 sets per pen). These samples were then weighed and then stored in -20⁰c freezer ready for collection and further processing, including: being

freeze dried and ground before measurement of zinc and titanium dioxide content. Blood serum samples were also collected post mortem, alongside excreta samples from litter for determination of zinc concentration. All samples were stored appropriately until collected.

There were no significant interactions between treatments on bird performance.

# Study objective

This study was designed to compare the zinc digestibility of 50% premix diets with and without insect larvae in comparison to a 100% premix diet in 4 week-old broilers, using the linear regression method.

Ethical approval to undertake this study was sought from the Nottingham Trent University School of ARES ethical review group, and granted approval logged as ARE1892268

# 5.2 Method

## 5.2.1 Husbandry conditions

Birds were sourced from PD Hook Cote hatchery, Oxford, from a flock aged 46 weeks. Birds were feather sexed on day of hatch and collected by NTU personnel to reduce travel stress. More birds than needed were be ordered so that poor birds were not allocated to trial pens. The birds used for this trial were reared from D0 on commercial broiler chick crumb on site until the start date of this trial. This trial started at D29 with 24 male Ross 308 chickens which were placed into pens in groups of 4. Feed and water were available *ad libitum*, with care taken to ensure the birds ate and drank as soon as possible. Feed was available from 30cm feed troughs in each pen, and water from nipple drinker lines with two nipples per pen. The lighting regimen was 23 hours light to 1 hour dark on D1 increasing by an hour of darkness a day until D6. 6 hours of dark (22:00-24:00 and 02:00-06:00) was then maintained for the remainder of the study (see Table 1). Temperature was set at 32°C on day of hatch and reduced by approximately 1°C per day. Temperatures were recorded at bird head height from two positions in the room with one thermometer positioned adjacent to the door and the other positioned in the far corner under an outside vent. Health checks were made twice daily, and heating and ventilation adjusted depending on bird behaviour. Ventilation was adjusted to maintain room temperature and humidity, while preventing ammonia build up.

On day 29 birds were weighed and marked up into sets ( 2 birds per set: 1 set of blank and 1 set with blue heads), with a total of 4 birds per pen, before allocation to 6 mesh sided pens (80cm*80cm) with wood shavings as litter substrate. The birds were allocated and zinc diet as per table 5.

Table 1: Lighting regime for the study FB01

| Day | Hours of light | Hours of dark |
| --- | --- | --- |
| 1 | 23 | 1 |
| 2 | 22 | 2 |
| 3 | 21 | 3 |
| 4 | 20 | 4 |
| 5 | 19 | 5 |
| 6 | 18 | 6 |
| 7 to 32 | 18 | 6 |

## 5.2.2 Diet formulation and manufacture

Birds were fed a commercial chick starter diet from d0-d28 They were then transferred onto one of 3 mash diets until d32. Diet formulations are presented in Table 2. Diets were mixed in house using a ribbon mixer. Diets were homogenized by mixing before analysis commenced and subsampled for zinc content. Dry micro ingredients were mixed in a bag by hand for 5 minutes whilst the dry macros were mixed for 5 minutes in the ribbon mixer, these were then mixed together in the ribbon mixer for another 5 minutes before the addition of the soya oil, then for a further 10 minutes to ensure homogeneity. Titanium dioxide was added to all diets at 5g/kg inclusion as an inert marker for digestibility measures. Grab samples were taken immediately post mixing for diet analysis to confirm correct manufacture.

Table 2: Dietary treatment formulations for FB01(g/kg)

| **Raw Material** | **Diet A** | **Diet B** | **Diet C** |
| --- | --- | --- | --- |
| Wheat | 680.2 | 680.2 | 679.2 |
| Soybean meal | 252.1 | 252.1 | 251.1 |
| Soya oil | 37.1 | 37.1 | 37.1 |
| Salt | 2.4 | 2.4 | 2.4 |
| Limestone | 8.2 | 8.2 | 8.2 |
| Dicalcium Phos | 4.8 | 4.8 | 4.8 |
| Sodium Bicarbonate | 2.0 | 2.0 | 2.0 |
| Lysine HCL | 2.4 | 2.4 | 2.4 |
| DL-Methionine | 2.6 | 2.6 | 2.6 |
| Threonine | 1.2 | 1.2 | 1.2 |
| Vitmin premix | 2.0 | 2.0 | 4.0 |
| Insects | - | 27.0 | - |
| Titanium dioxide | 5.0 | 5.0 | 5.0 |

Table 3: Analysed proximate content of treatment diets for study FB01

| Diet | A | B | C |
| --- | --- | --- | --- |
| Zn (mg/kg) | 33 | 50 | 49 |

## 5.2. 3 Treatment schedule / randomisation plan

The 3 dietary treatments are shown in Table 4 for the varying protein sources.

Table 4: Dietary treatment IDs for FB01

| **Treatment** | **Protein Source** |
| --- | --- |
| A | 50% Premix |
| B | 50% Premix plus Insects |
| C | 100% Premix |

One replicate was 2 broilers, with two replicates per pen, meaning a total of 4 broilers per pen. Birds were weighed in replicates, marked (blank and blue heads), allocated to pens and dietary treatments on d29. Diet allocation is shown in Table 5. There were 2 pens per diet making a total of 4 replicates per treatment. Diet allocation was randomised by block (2 blocks total).

Table 5: Diet allocation for FB01

| **Pen** | **Diet** |
| --- | --- |
| 1 | A |
| 2 | B |
| 3 | C |
| 4 | A |
| 5 | B |
| 6 | C |

## 5.3 Observations during the study

### 5.3.1 Environmental Control

Bird observations were used to monitor the environment and if the birds appeared uncomfortable, the temperature and/or ventilation was altered accordingly.

### 5.3.2 Health and Condition

Birds were observed twice daily during the trial and any observations related to health recorded in a trial diary. Any dead birds were weighed and reasons recorded if culled.

### 5.3.3 Feed Intake Measurement

Individual weighed and labelled bags of feed were prepared containing 4kg of feed on d29 for each pen. Each pen of birds was fed from their designated bag for the trial period. Uneaten food in the troughs on d32 was tipped back into the labelled feed bag for each pen, taking care to remove droppings and shavings. Total feed eaten was calculated as the difference between remaining feed in the bag and the amount weighed into the bag for the trial period, and feed intake expressed as grams of feed eaten per bird.

### 5.3.4 Body Weight Gain

Birds were weighed by replicate and pen (2 replicates per pen) on d29 to exclude outliers from the subsequent study and then weighed by pen on d32. Bodyweight gain is expressed as grams gained per bird during the trial period.

### 5.3.5 Sample Collection

On d32, birds were sequentially fed fresh diet for a minimum of 30 minutes to ensure gut fill before culling. All 4 birds per pen (2 sets, 2 birds per set) were then sequentially culled by cervical dislocation before bloods were taken by opening up the birds, cutting the aorta and collecting the blood using a syringe and placing it in a blood tube ready for it to be spun down to separate the serum. The serum was then collected into 7ml tubes using pasture pipettes.

Ileal digesta was also collected by removing the small intestine from Meckel’s diverticulum to the ileal-caecal junction. The digesta was removed by gentle digital pressure and pooled into one pot per set, per pen (E.g. Pen 1, Set A). Digesta was stored at -20^o^C prior to freeze drying and grinding. Ground digesta was analysed for TiO_2_ content and zinc content.

Excreta samples were manually collected into labelled trays, on a per pen basis. These samples were weighed prior to drying in the oven, weighed when removed and ground before being stored, ready for collection/analysis.

### 5.4 Laboratory techniques

*5.4.1 Titanium dioxide*

Titanium dioxide content of diets and digesta was measured using the method of Short *et al.* (2006). Briefly, 0.3g of freeze dried digesta were ashed at 650^o^C in a ceramic crucible then digested with 15ml 7.4M H_2_SO_4_ until all the TiO_2_ was dissolved. The acid digest was then diluted with water and filtered into a 100ml volumetric flask. 10ml of hydrogen peroxide was added and the flasks made to volume with water. Flasks were mixed well, and absorbance measured at 410nm with TiO_2_ content calculated using a standard curve of known TiO_2_ concentrations.

*5.4.2 Zinc analysis*

Zinc content of blood, excreta, digesta and larvae was carried out using ICP-MS.

Using the TiO_2_ measurements, the zinc results were used to calculate apparent zinc digestibility using the following equation:

1-(zinc_dig_ * marker _feed_) / (zinc _feed_ * marker _dig_)

Where

zinc_dig_ represents the zinc content of the digesta

marker_feed_ represents the titanium concentration in the diet

zinc_feed_ represents the zinc concentration in the diet

marker_dig_ represents the titanium dioxide concentration in the digesta

The determined apparent digestible zinc content of the diets was regressed against rate of inclusion of each protein source. The linear regression was then extrapolated to a rate of inclusion of 100% (or 1000g/kg) 50% Premix, 50% Premix with insects, or 100% Premix, to give a figure for apparent digestible content for each zinc measured. Dividing this figure by the total content of the specific zinc in the protein sources gave a coefficient of apparent zinc digestibility.

5.5 Statistical analysis of data

Statistical analysis was carried out using SPSS v.28. to determine interactions between the analysed factors, and one-way ANOVA to test the equality of the means to investigate the effect of dietary treatment on performance.

# 6 Results

### 6.1 Environment

No environmental anomalies occurred during this trial.

### 6.2 Health and Condition

No birds died whilst on the experimental diets (d29-32)

### 6.3 Bird Uniformity

Birds were weighed into individual pens, 2 sets per pen, 4 birds total (Marked and unmarked) on d29. Mean start weights for each treatment are shown in Table 6. There was no significant difference in start weight across the dietary treatments

Table 6: Start weights for chicks in trial FB01 S.E

| Treatment | d29 BW/bird (g) S.E |
| --- | --- |
| A | 1127.313 (19.237) |
| B | 1117.313 (12.663) |
| C | 1126.613 (8.063) |
| p value | 0.861 |

### 6.4 Performance

Table 7 shows that were no significant interactions between treatments in FI, BWG and FCR. Individual pen bird weights for the start and finish of the study period are shown in Appendix 2.

Table 7: Performance data from d29 to d32 in study FB01 S.E

| Treatment | d29-32 FI/bird (g) S.E | d29-32 BWG/bird (g) S.E | D29-32 FCR S.E |
| --- | --- | --- | --- |
| A | 502.8 (28.3) | 265.062 (38.563) | 1.954 (0.391) |
| B | 540.688 (88.413) | 289.688 (1.838) | 1.865 (0.293) |
| C | 541.863 (26.888) | 268.837 (9.687) | 2.015 (0.027) |
| p value | 0.860 | 0.740 | 0.933 |

### 6.5 Apparent digestible zinc content of the treatments

| Zinc source | Blood serum Zn (ppm) | Digestible Zn content (g/kg diet) |
| --- | --- | --- |
| Premix at 50% commercial standard | 25.1 | 3.6c |
| 50% Premix plus Nanozinc enriched Insects | 21.6 | 6.5a |
| Premix at commercial standard level | 25.7 | 6.4b |
|  |  |  |
| SEM | 2.75 | 3.5*10^-5 |
| P | 0.589 | <0.001 |

Diet B had significantly the highest level of digestible Zn content. Blood serum level of Zn was not significantly affected by the diet.

### 6.6 Coefficients of Digestibility

| Diet | d32 Zn digestibility |
| --- | --- |
| Diet A | 0.999867 |
| Diet B | 0.999854 |
| Diet C | 0.99987 |
|  |  |
| SEM | 5.58*10^-6 |
| P | 0.242 |

The diet did not significantly altered Zn digestibility coefficient of broilers.

## Bird start and end weight for trial period (FB01)

| **Pen** | **Diet** | **D29 Bird Wt (g)** | **D32 Bird Wt (g)** |
| --- | --- | --- | --- |
| 1 | A | 1146.6 | 1450.2 |
| 2 | B | 1130.0 | 1417.8 |
| 3 | C | 1118.6 | 1377.7 |
| 4 | A | 1108.1 | 1334.6 |
| 5 | B | 1104.7 | 1396.2 |
| 6 | C | 1134.7 | 1413.2 |
